# Single copy optogenetic system for *Streptomyces*

**DOI:** 10.1038/s41598-025-27850-9

**Published:** 2025-12-15

**Authors:** Airi Watanabe, Ryuta Noya, Rio Yamada, Hideaki Takano

**Affiliations:** Life Science Research Center, College of Bioresource Sciences, Nihon University, 1866 Kameino, Fujisawa, 252-0880 Japan

**Keywords:** Optogenetics, *Strepomyces griseus*, LitR, Expression system, Applied microbiology, Bacterial genetics

## Abstract

LitR is a blue-green light-sensing transcriptional regulator that uses coenzyme B_12_ as a chromophore. In this study, we developed a genome-integrative light-inducible expression (iLiEX) system in *Streptomyces griseus* NBRC 13350, a Gram-positive bacterium that produces streptomycin. The system incorporates LitR, transcriptional amplification module T7 RNA polymerase, and a serine integrase. Using iLiEX, we achieved light-dependent overproduction of catechol-2,3-dioxygenase and β-glucuronidase (GUS) at levels comparable to those from a high-copy plasmid. Notably, GUS activity was 39-fold higher than with the constitutively strong ermE* promoter. The iLiEX system was also functional in *S. coelicolor*, *S. lividans*, *S. albus* J1074, and *S. avermitilis*. We improved iLiEX in two key ways: by optimizing the ribosome-binding site of *T7 RNA polymerase* to increase expression, and by introducing the *T7 lysozyme* gene to reduce leaky transcription. The system’s versatility was improved by shortening the *T7* promoter from 89 to 44 bp. For simple visualization on agar plates, light-dependent overexpression of fluorescent proteins, a chromogenic protein, and a brown pigment synthesis enzyme was demonstrated. High-level production of secreted enzymes, including laccase and transglutaminase, was also confirmed. Overall, we developed a single-copy light-inducible overexpression system with broad functionality across multiple *Streptomyces* species.

**Supplementary Information:**

The online version contains supplementary material available at 10.1038/s41598-025-27850-9.

## Introduction

*Streptomyces* are Gram-positive soil-dwelling bacteria with a strong ability to decompose organic matter in dead plants. Their saprophytic nature plays a key role in nutrient cycling within soil ecosystems^1,2^. *Streptomyces* exhibits two notable features: (i) complex morphological differentiation resembling that of fungi, and (ii) the production of diverse secondary metabolites, many with bioactive properties^1,2^. Their life cycle includes spore germination, elongation of the filamentous substrate mycelia, and development of aerial mycelium, culminating in mature spore formation. The latter two stages represent morphological differentiation. During this phase, *Streptomyces* species produce secondary metabolites such as antibiotics, anticancer drugs, and immunosuppressants^3^. Numerous biosynthetic pathways and unique enzymatic reactions have been characterized^2,4,5^. In addition, *Streptomyces* are known to produce various industrial enzymes, including xylanase^6^, endoglucanase^7^, transglutaminase^8^, protease^9^, and phospholipase D^10^, which contribute to their saprophytic properties.

Genetic manipulation methods are widely used in *Streptomyces* research to investigate the molecular mechanisms underlying morphological differentiation, secondary metabolite biosynthesis, and the heterologous production of secondary metabolites and recombinant proteins^5^. DNA vectors used in genetic studies of *Streptomyces* include: (i) self-replicating, high-copy-number plasmids^11,12^, and (ii) chromosome-integrative (Int) vectors^13,14^. High-copy plasmids enable high expression owing to gene dosage effects. However, they suffer from low transformation efficiency and narrow host range via conjugative transfer. Int vectors encode genes for serine- or tyrosine-type integrases derived from bacteriophages, allowing efficient and stable integration into the genome^15,16^. These Int vectors were utilized for heterologous expression of large secondary metabolite gene clusters. The expression level of transgenes in Int vectors is generally lower than that achieved with high-copy-number plasmids because of their single-copy status within the genome. Conversely, transformation efficiency via conjugative transfer is exceptionally high when mediated by phage integrases. Thus, these two vector types offer complementary strengths and limitations.

Chemically inducible expression systems have been used to control cellular functions in genetically modified *Streptomyces*. In particular, systems inducible by the antibiotic thiostrepton^17^ or the amide compound ε-caprolactam^18^ have been employed for many years. However, they have limitations, including the emergence of drug-resistant strains and the use of non-natural compounds. More recently, several studies have reported new induction systems, including biodegradable induction systems that can be controlled using plant sugars (xylose^19^, rhamnose^20^, and cellobiose^21^) and compound (cumate; 4-isopropylbenzoic acid)^22^. However, chemical inducers are associated with the following difficult-to-solve problems: (i) disposal of inducers, (ii) changing the medium composition by adding the inducers themselves, and (iii) irreversible control of the gene switch.

We recently developed a high-copy *p*lasmid-based *l*ight-*i*nducible *ex*pression system (pLiEX) in the streptomycin-producing *Streptomyces griseus* NBRC 13350^23^. Using pLiEX, we successfully achieved mass-production of both cytoplasmic and secretory enzymes. Compared to existing light-sensing systems used in optogenetics, such as the Magnet system^24^, pLiEX can be activated by extremely weak blue to green light and responds to light intensities found in everyday environments. Consequently, pLiEX system does not require specialized or expensive equipment and is sufficient for attaching a commercially available and inexpensive LED light source to an incubator using a magnet. To the best of our knowledge, this is the first report on a practical optogenetic tool for *Streptomyces*. However, a limitation of pLiEX is its narrow host range, as it does not function in the model strains *S. lividans* and *S. coelicolor*.

This study aimed to overcome the narrow host range of the plasmid-based pLiEX system, a major limitation to its broader application. *Streptomyces* species produce a wide range of unique secondary metabolites that vary significantly between species. Establishing an inducible high expression system tailored to each *Streptomyces* species will support both the efficient production and discovery of valuable substances. Achieving strong expression from a single copy using a genome-integrated vector with high transformation efficiency via conjugation without relying on a high-copy plasmid is expected to be a versatile technology in both basic and applied research fields concerning *Streptomyces* and related bacterial genera. Here, we report an improved single-copy LiEX system that exhibits broad functionality within the genus *Streptomyces* by combining LitR with the T7 RNA polymerase as a transcriptional amplification machinery.

## Results

### Development of a genome-integrative light-inducible expression system

Recently, we developed a plasmid-type light-inducible expression system (pLiEX) using *Streptomyces griseus* NBRC 13350 as a host. In our previous study, a one plasmid-type pLiEX system produced large quantities of recombinant proteins in response to weak blue-green light^23^. In this system, light stimuli are received by LitR, a coenzyme B_12_-based MerR-type photosensory regulator derived from *S. coelicolor* A3(2), and activated LitR promotes transcription of *litS* promoter^25,26^. The RNA polymerase holoenzyme containing σ^LitS^ directs transcription from the *crtE* promoter. Consequently, the Gene of Interest (GOI) under the control of *crtE*p was specifically expressed under light conditions. The key feature of this system is its simple one-plasmid design, which enables light-dependent mass production of a group of valuable enzymes by introducing a foreign gene into pLit19. However, pLiEX works only in a few *Streptomyces* spp.; it does not work in model strains such as *S. coelicolor* and *S. lividans* (Supplementary Fig. S1). In addition to the limited host range, the one-plasmid-type pLit19 also has two further drawbacks: (i) a restriction on the DNA size that can be inserted into the plasmid (as far as we investigated, the maximum is 8 kbp^27^), and (ii) a high frequency of plasmid loss in transformants when selective antibiotics are not used (with 98% plasmid loss after five culture passages^28^).

**Fig. 1:**
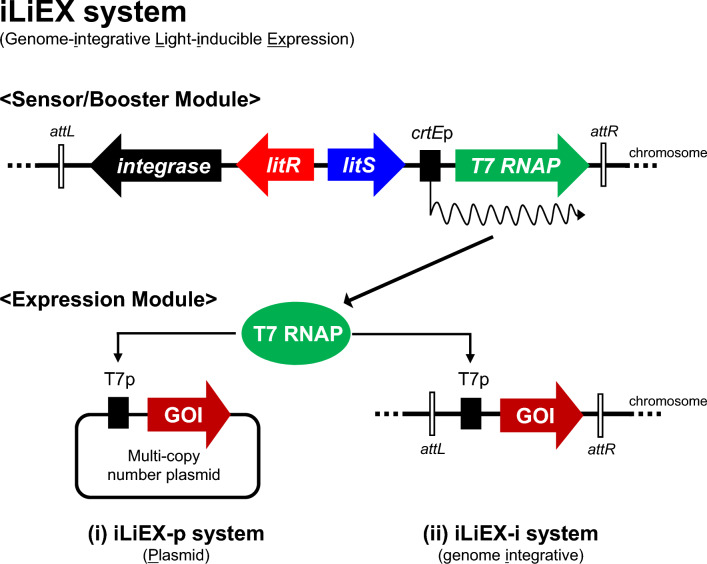
Schematic representation of two iLiEX systems. Two light-inducible expression (iLiEX) systems have been described: (i) a high-copy plasmid-based system, iLiEX-p, and (ii) a single-copy integrative system, iLiEX-i. A common feature of both systems is the genomic integration of a light sensor and a transcriptional amplification module. The key difference lies in the location of the gene of interest (GOI) expression module; it is introduced into a high-copy plasmid in iLiEX-p and integrated into the genome in iLiEX-i. LitR undergoes a functional change upon stimulation with relatively weak blue-to-green light, resulting in the positive regulation of LitS transcription. Subsequently, the RNA polymerase holoenzyme containing σ^LitS^ transcribes the *T7RNAP* gene. Consequently, T7RNAP specifically transcribes the *T7* promoter, leading to GOI expression. T7RNAP then repeatedly synthesizes mRNA from the *T7* promoter, enabling high-level GOI expression.

To broaden the host range, we developed two modified LiEX variants (Fig. 1): (i) an *i*ntegrative *iLiEX*-*p*lasmid reporter system (iLiEX-p) and (ii) a fully *i*ntegrative *iLiEX* reporter system (iLiEX-i). In the iLiEX-p, the light sensor and transcriptional amplification modules were integrated into the genome, while the GOI was placed downstream of the *T7*p on a self-replicative pIJ101-based high-copy number plasmid. In the iLiEX-i system, all the modules, including the light sensor, transcription amplification system, and GOI, were inserted into the genome. In both systems, the light-sensing module was integrated into the genome. To compensate for the reduced gene dosage in high-copy number plasmids, we used T7 RNA polymerase (T7RNAP), a high-amplification machinery, in the transcriptional circuit.

T7RNAP is a DNA-dependent RNA polymerase composed of a single subunit with 883 amino acids, derived from *E. coli*-infecting T7 bacteriophages. It specifically recognizes a 17 bp promoter sequence (5′-TAATACGACTCACTATA-3′), and transcribes at a rate approximately eight times faster than that of the multi-subunit *E. coli* RNA polymerase^29,30^. Because of its high specificity and transcription efficiency, it has been safely used for several decades in recombinant protein production systems in *E. coli*^31^ and, more recently, in mRNA vaccines to synthesize large amounts of mRNA encoding antigenic proteins via in vitro transcription^32,33^.

### High production of XylE and GUS with iLiEX-p and iLiEX-i systems

**Fig. 2:**
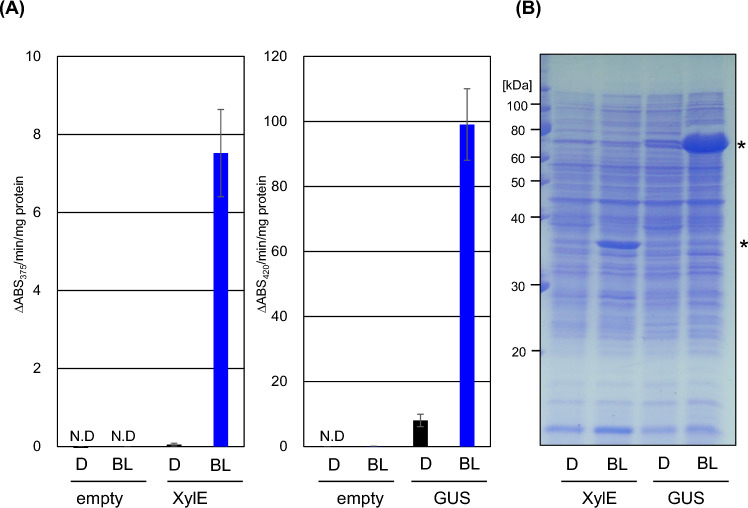
High production of XylE and GUS enzymes using the iLiEX-p system. (**A**) *S. griseus* transformants harboring the iLiEX-p system with GOIs encoding *xylE* or *gus* were cultured with shaking for 48 h under dark (D) or blue light (BL; center wavelength: 450 nm; 3 μmol s^−1^ m^−2^). Enzymatic activities of XylE and GUS in the cell-free extracts were calculated as the change in absorbance (ABS) at 375 and 420 nm per minute per milligram of total protein (ΔABS375 nm/min/mg or ΔABS420 nm/min/mg), respectively. Data are represented as mean ± standard deviation (SD) from three independent experiments (*n* = 3). N.D. not detected due to the lower limit of XylE and GUS activity. (**B**) Total intracellular proteins from the transformants were separated by sodium dodecyl sulfate–polyacrylamide gel electrophoresis (SDS-PAGE) and stained with Coomassie Brilliant Blue R-250 (CBB). Asterisks (*) indicate the band corresponding to XylE or GUS, identified based on their predicted molecular mass. The full-length, unprocessed gel image is shown in Supplementary Fig. S2. The samples derive from the same experiment, and the gels were processed in parallel.

First, we analyzed the function of the plasmid-based iLiEX-p system. In this system, the light sensor (*litR*・*litS*) and transcriptional amplification (*T7RNAP*) modules were integrated into the φBT1 chromosomal attachment site (*attB*_ϕBT1_) of *S. griseus* via the activity of φBT1 serine integrase. The GOI was introduced into the pIJ702-based high-copy-number plasmid under the control of the T7 promoter. Two reporter enzymes, catechol 2,3-dioxygenase^34^ (XylE) from *Pseudomonas putida* and β-glucuronidase (GUS)^35^ from *E. coli* K-12 optimized for codon usage frequency in *S. coelicolor*, were used to evaluate the system. Transformants were grown under blue light and dark conditions, and enzyme activity was measured using a chromogenic substrate (see Materials and Methods). As shown in Fig. 2A, enzyme activity was low under dark conditions but increased significantly under blue light. To evaluate intracellular protein levels, total protein in the cell lysate was separated by sodium dodecyl sulfate–polyacrylamide gel electrophoresis (SDS-PAGE) and stained with Coomassie Brilliant Blue R-250 (CBB). As shown in Fig. 2B and Supplementary Fig. S2, both enzymes were most abundant in the cytoplasm when the transformants were cultured under light conditions. The production level of XylE in the iLiEX-p system was similar to that in the one-plasmid-type pLiEX, indicating that the iLiEX-p system has a similar ability as pLit19 in pLiEX. The load on the iLiEX-p system was low, because the gene introduced into the plasmid of the iLiEX system was only a GOI. Therefore, compared to pLiEX, this system can be used to introduce large DNA fragments containing multiple GOIs.

**Fig. 3:**
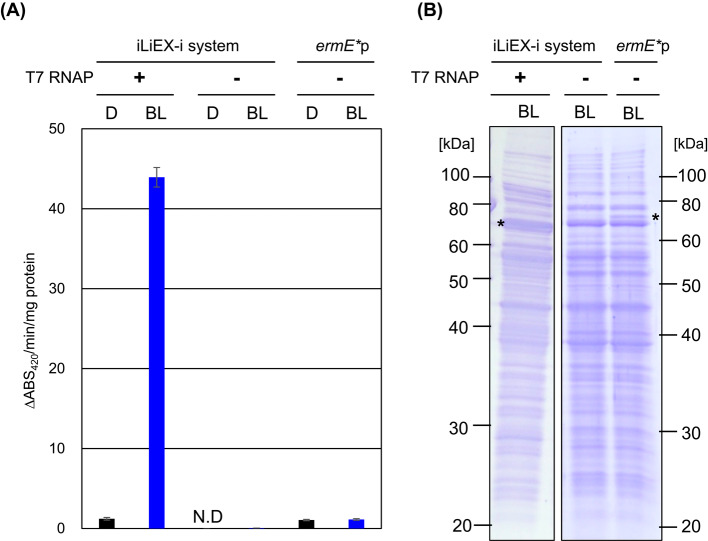
High production of GUS with iLiEX-i system. (**A**) Three types of *S. griseus* transformants were cultured with shaking for 48 h under dark (D) and blue light (BL): (i) harboring the iLiEX-i system, (ii) harboring the iLiEX-i system without *T7 RNAP*, and (iii) harboring ermE*p as a constitutive strong promoter (control for a non-iLiEX system). GUS was used as a reporter enzyme in all cases. GUS activity was measured and is presented as described above. Data are represented as mean ± SD from three independent experiments (*n* = 3). N.D. not detected due to the lower limit of GUS activity. (**B**) Total intracellular proteins were separated by SDS-PAGE and stained using CBB. The asterisk (*) indicates the band corresponding to GUS. The original, unprocessed images of the full-length gel are included in Supplementary Fig. S3 and S4. The samples derive from the same experiment, and the gels were processed in parallel.

Subsequently, we constructed the iLiEX-i system, where all components (*litR*, *litS*, *crtE*p, and *T7RNAP)* were inserted into the *attB*_ϕBT1_ site, and T7p-*gus* was inserted into the φC31 chromosomal attachment site (*attB*_ϕC31_) of *S. griseus*. As shown in Fig. 3, and Supplementary Fig. S3 and S4, this transformant exhibited low GUS activity in the dark. However, it showed extremely high GUS activity in response to blue light illumination, with an activity level of approximately 50% that of iLiEX-p. We then compared it with non-T7RNAP iLiEX-i (*litR-litS-crtE*p) as a native light-inducible promoter; iLiEX-i showed 922 times higher activity than the non-iLiEX system. We also compared it with the constitutive ermE* promoter (ermE*p), a widely used strong promoter; iLiEX-i showed 39 times higher activity than ermE*p. In the absence of *T7RNAP*, no GUS activity was detected in transformants carrying T7p-GUS. These results demonstrate that extremely high expression of a single-copy GOI can be achieved using this system.

### iLEX-i system functions across *Streptomyces* strains

**Fig. 4:**
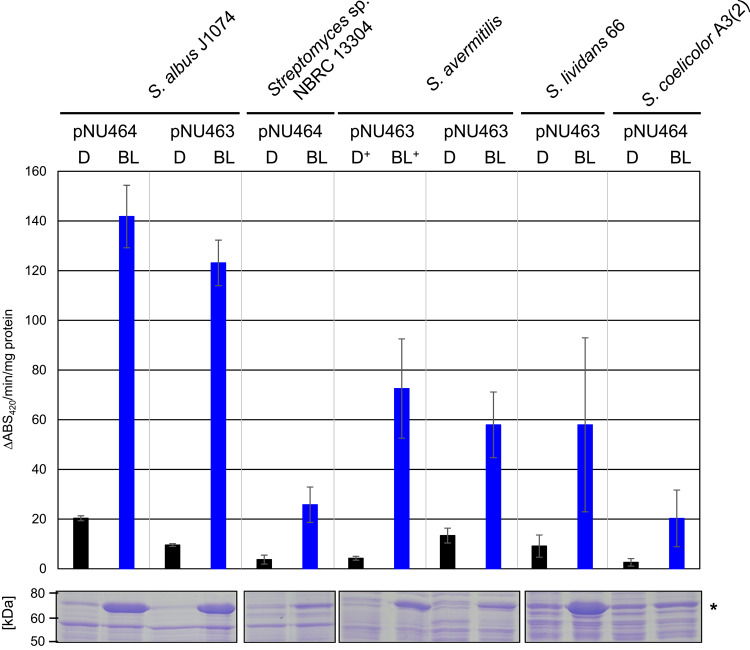
Functionality of the iLiEX-i system in *Streptomyces* model strains. Transformants of *S. albus* J1074, *Streptomyces* sp. NBRC 13,304, *S. avermitilis* MA-4680, *S. lividans* 66, and *S. coelicolor* A3(2) harboring the iLiEX-i system with *gus* were cultured with shaking for 48 h under dark (D) and blue light (BL) conditions. + indicates the addition of AdoB_12_ (final concentration 10 μM) to the culture media. GUS activity was quantified and marked as described above. The genome-integrative *attB* sites used in each strain are indicated as pNU464 (φBT1) or pNU463 (φK38-1). Data are represented as mean ± SD from three independent experiments (*n* = 3). N.D. not detected due to the lower limit of GUS activity. Total intracellular proteins were separated by SDS-PAGE and stained using CBB (shown at the bottom). The asterisk (*) indicates the band corresponding to GUS. The original, unprocessed images of the full-length gel are included in Supplementary Fig. S5 and S6. The samples derive from the same experiment, and the gels were processed in parallel.

To investigate whether the iLEX-i system functions broadly in *Streptomyces*, the following model strains were used as hosts: *S. albus* J1074^36,37^, a cephamycin-producing *Streptomyces* sp. NBRC 13304^38^, *S. avermitilis* MA-4680^39^, *S. lividans*^40^, and *S. coelicolor* A3(2)^41^. GUS was used as a reporter enzyme, and the reporter vector was integrated into the *attB*_φC31_ site of the genome using a φC31-type integration vector (pTYM19). To introduce Booster into the genome, φK38-1 (pNU463) or φBT1 integrase vector (pNU464) was used, as their integration frequencies vary depending on the strain. As shown in Fig. 4, and Supplementary Fig. S5 and S6, light-dependent GUS activity was observed in all five strains, with high productivity observed in *S. albus* J1074, *S. avermitilis*, and *S. lividans*. GUS production levels in *Streptomyces* sp. NBRC 13,304, and *S. coelicolor* A3(2) were not high, but a protein band corresponding to GUS was detected (Fig. 4). In *S. avermitilis*, light-dependent carotenoid production regulated by LitR and LitS was observed only when coenzyme B_12_ was externally added to the culture medium. Therefore, we analyzed GUS production under two conditions: with and without exogenous B_12_ supply. As a result, supplying B_12_ reduces GUS activity under dark conditions (Fig. 4). Conversely, supplying B_12_ increased the GUS activity under light conditions. This suggests that the exogenous supply of B_12_ enhances the repressor and activator functions of LitR under dark and light conditions, respectively. Overall, these results strongly suggested that the single-copy iLiEX-i system is broadly functional across *Streptomyces* spp.

### Optimization of ribosome-binding site (RBS) and translational initiation (start) codon for *T7RNAP*

**Fig. 5:**
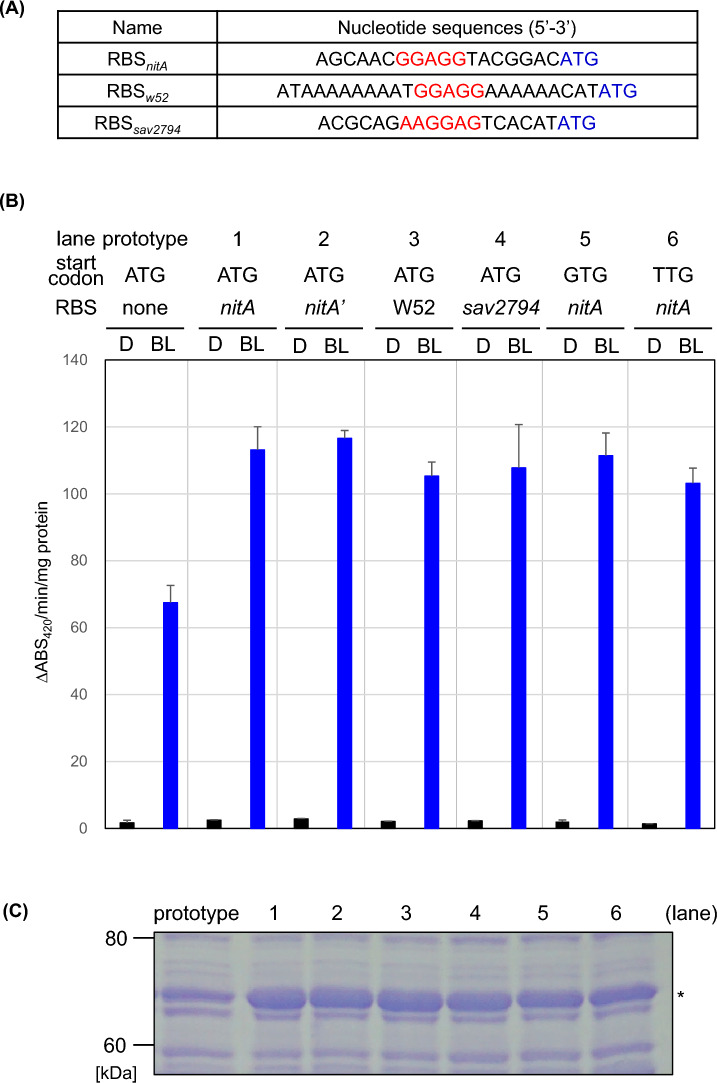
Optimization of the ribosome-binding site (RBS) and start codon of *T7RNAP*. (**A**) Nucleotide sequences of RBSs (RBS_*nitA*_, RBS_*W52*_, and RBS_*sav2794*_) are shown in red along with their neighboring regions. (**B**) iLiEX-i transformants of *S. griseus* harboring *T7RNAP* with four types of RBSs and/or three types of start codons were grown in shaking culture for 48 h under dark (D) and blue light (BL) conditions. GUS activity was quantified and marked as described above. Prime (‘) for lane 2 means insertion of the *Nde*I site between RBS and start codon. Data are represented as mean ± SD from three independent experiments (*n* = 3). (**C**) Total intracellular proteins were separated by SDS-PAGE and stained using CBB (shown at the bottom). The asterisk (*) indicates the band corresponding to GUS. The original, unprocessed image of the full-length gel is included in Supplementary Fig. S7.

The prototype vector used in the above analysis lacked the RBS of the *T7 polymerase* gene in our original design. Therefore, we examined the RBS and start codons to improve protein productivity further. Six booster vectors were constructed, including three well-recognized RBS sequences (RBS_*nitA*_, RBS_*W52*_, and RBS_*sav2794*_, Fig. 5A)^18,42,43^ and three start codon types (ATG, GTG, and TTG). Their effects were evaluated based on GUS activity. As shown in Fig. 5B, we confirmed that the GUS productivity of the six vectors was approximately 1.5 to 2 times higher than that of the prototype vector, and the GUS activity reached a level similar to that of the plasmid-type iLiEX-p system. The GUS protein bands in lanes 1–6 were thicker than those in the prototype (Fig. 5C and Supplementary Fig. S7). The highest GUS activity was detected when RBS_*nitA*_, derived from the nitrile hydratase gene of *Rhodococcus rhodochrous*^44^, a genus related to *Streptomyces*, was used. RBS_*nitA*_ (5′-AGCAACG*GGAGG*TACGGAC*ATG*-3′; RBS_*nitA*_ and the start codon are shown in italics) is a well-recognized RBS in *Streptomyces* and is utilized in the epsilon-caprolactam-inducible *nit* and our pLiEX systems, making it suitable for high-level production in heterologous *Streptomyces*. In contrast, there were no significant differences between the three start codons. Thus, the introduction of RBS_*nitA*_ improved the functionality of the iLiEX-i system.

### Repression of leakage transcription by introducing *T7 lysozyme* gene

**Fig. 6:**
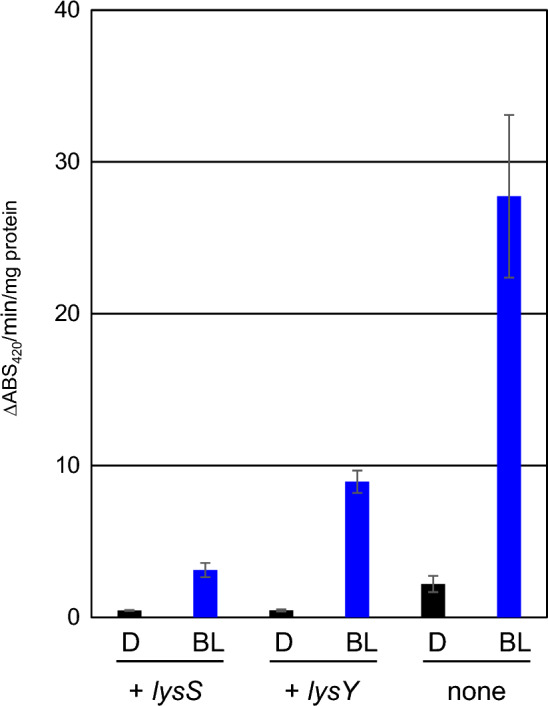
Suppression of leakage expression by *lys* genes. iLiEX-i transformants harboring *gus* with the wild-type *lysS* or the mutant *lysY* were cultured with shaking for 48 h under dark (D) and blue light (BL) conditions. GUS activity was measured and marked as described above. Data are represented as mean ± SD from three independent experiments (*n* = 3).

We successfully optimized the RBS; however, low GUS activity was detected under dark conditions (Fig. 5B). We then attempted to prevent basal-level transcriptional leakage under non-inducing conditions. In the pET system, a T7RNAP-based recombinant protein expression system in *E. coli*, two types of T7 lysozyme genes, *lysS* and *lysY*, were used to avoid leakage of expression by T7 polymerase^45^. Wild-type LysS (151 aa) has two functions: (i) N-acetylmuramoyl-L-alanine amidase activity for cell wall lysis, and (ii) inhibition of T7RNAP function via protein–protein interactions. LysY is a single amino acid substitution mutant of LysS that has lost amidase activity, but retains its inhibitory activity against T7RNAP. We examined the effects of *lysS* and *lysY* on leakage gene expression in *S. griseus*. The introduction of *lysS* or *lysY* into the iLiEX-i system decreased GUS induction activity under both dark and light conditions (Fig. 6). However, the basal expression was significantly reduced under dark conditions. The fold change in response to light, which was 12 in the strain without *lys*, increased to 19 with the introduction of *lysY*. Therefore, we confirmed that LysY functions in *S. griseus* and can be used to prevent the leakage of T7RNAP, thereby enhancing the function of the iLiEX system.

### Shortening the T7 promoter

**Fig. 7:**
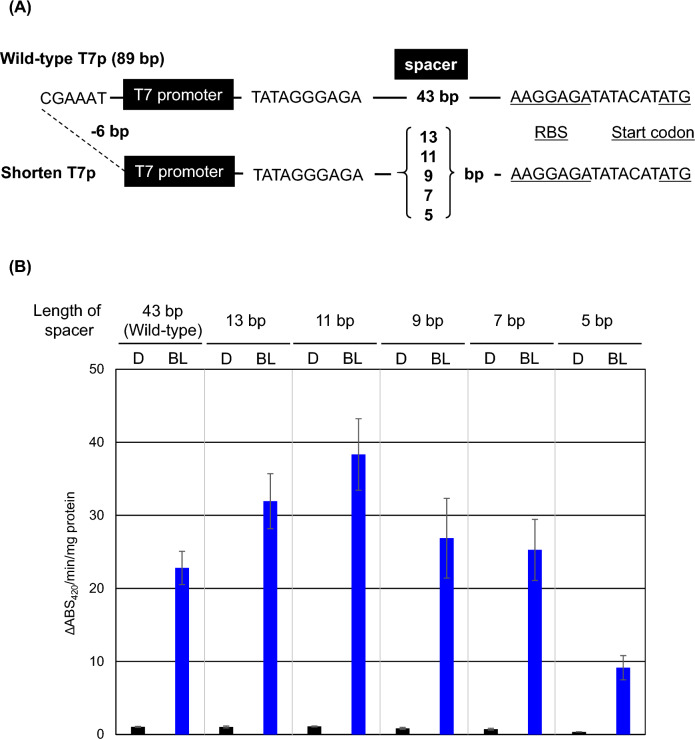
Trimming of T7 promoter. (**A**) A schematic representation of the T7 promoter is shown. The wild-type T7 promoter is 89 bp in length and includes an upstream region, a T7RNAP recognition site, a spacer region, and RBS. In the trimmed T7 promoter, the upstream region has been entirely removed, and the spacer region has been shortened. (**B**) iLiEX-i transformants with *gus*, harboring either the wild-type or trimmed T7 promoter, were cultured with shaking for 48 h under dark (D) and blue light (BL) conditions. GUS activity was quantified as described above. Data are represented as mean ± SD from three independent experiments (*n* = 3).

The wild type T7 promoter is 89 bp long, which increases the cost of synthesizing artificial DNA and oligonucleotides and has a significant environmental impact during the synthesis process. The T7 promoter consists of five elements: the upstream region, T7 promoter itself, transcriptional initiation region, spacer region, and RBS (Fig. 7A). We analyzed the 6 bp upstream region and the 43 bp spacer region, which were predicted to be non-essential, as candidates for trimming. The upstream region was entirely deleted and the spacer region was progressively truncated to 13, 11, 9, 7, and 5 bp. Transformants were then generated, and the function of the shortened T7 promoter was evaluated by measuring GUS activity. As shown in Fig. 7B, even with complete deletion of the upstream region, the transcriptional level remained unaffected and was comparable to that of the wild-type. Stepwise trimming of the spacer region to 13 bp and 11 bp resulted in a slight increase in GUS activity compared to the wild-type 43 bp spacer, whereas shortening to 9 bp and 7 bp yielded activity levels similar to those of the wild type. In contrast, shortening the length to 5 bp decreased GUS activity. These results indicated that the 7 bp spacer maintained transcriptional levels equivalent to those of the wild type. Overall, we successfully shortened the promoter length from 89 to 44 bp, which enhanced the versatility of the iLiEX system.

### Visualizing gene expression on agar plates

**Fig. 8:**
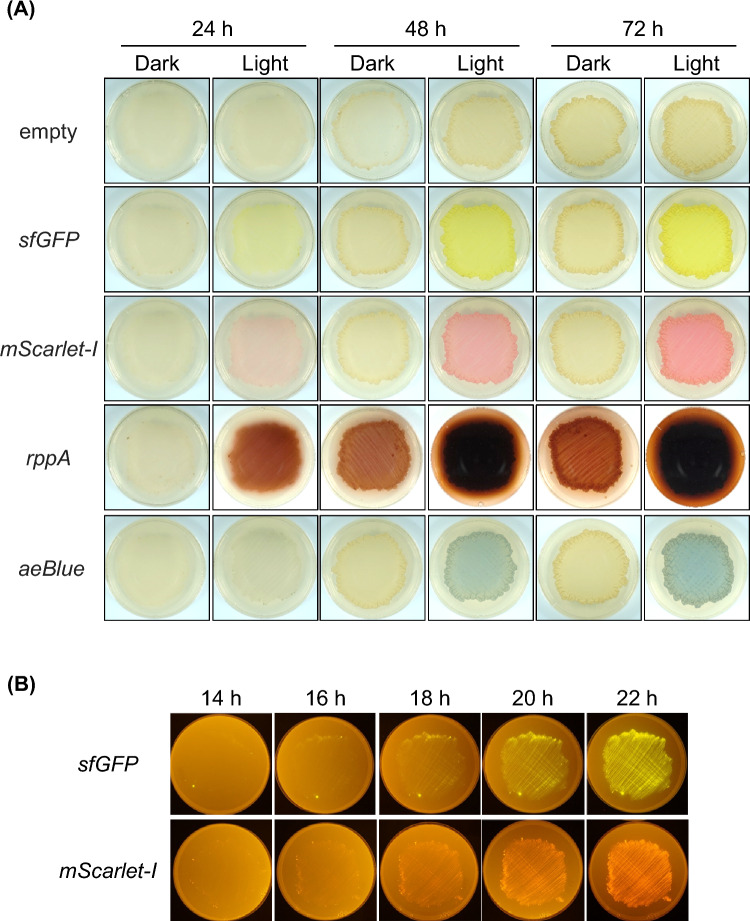
Visualizing gene expression on agar plates. (**A**) *S. griseus* transformants of iLiEX-i harboring GOI encoding *sfGFP* for green fluorescent protein, or *mScarlet-I* for red fluorescent protein, *rppA* for the brownish-red pigment flaviolin, and *aeBlue* for blue chromogenic protein were cultured on YMP-glucose solid medium for 72 h in the dark and blue light (BL, 3 μmol s^−1^ m^−2^) conditions. Under indoor white-light illumination (without excitation light), fluorescence and pigmentation was visually analyzed and photographed. (**B**) Upon irradiation with excitation light in a dark room, fluorescence from both sfGFP and mScarlet-I was analyzed every 2 h from 14 to 22 h during the early growth phase. The original, unprocessed image of photos is included in Supplementary Fig. S8.

GUS was used as a cytoplasmic reporter enzyme to evaluate the iLiEX-i system. However, the GUS assay involves time-consuming cell lysis steps. Furthermore, the accuracy of the GUS assay on a solid culture medium containing a high-cost chromogenic substrate is not high^35^. Therefore, we investigated the effectiveness of four markers for visualizing and evaluating the iLiEX-i system on a solid agar medium. We introduced marker genes into the iLiEX-i system and observed the fluorescence intensity and coloration generated by *Streptomyces* transformants grown on agar medium in response to blue light. As shown in Fig. 8A and Supplementary Fig. S8A, Superfolder green fluorescent protein (sfGFP)^46^, red fluorescent protein (mScarlet-I)^47^, and brownish-red pigment flaviolin, synthesized by the type III polyketide synthase RppA^48^, were detected after 24 h of incubation. The blue chromogenic protein aeBlue^49^ was detected after 48 h of incubation. Because these fluorescent proteins were observed to emit green and red fluorescence even under indoor white-light illumination (without excitation light), each protein was predicted to be overexpressed within the cell. We then observed gene expression within 24 h of incubation in the early growth phase because the analysis tended to be unstable owing to the low number of cells. When we analyzed the transformants by irradiation with excitation light in a dark room, we observed fluorescence from both sfGFP and mScarlet-I after 18 h of incubation (Fig. 8B and Supplementary Fig. S8B). This demonstrates that the application of visibility markers allowed the iLiEX-i system to detect gene expression with high sensitivity during the early growth phase on agar plate.

### High-level production of secreted enzymes

**Fig. 9:**
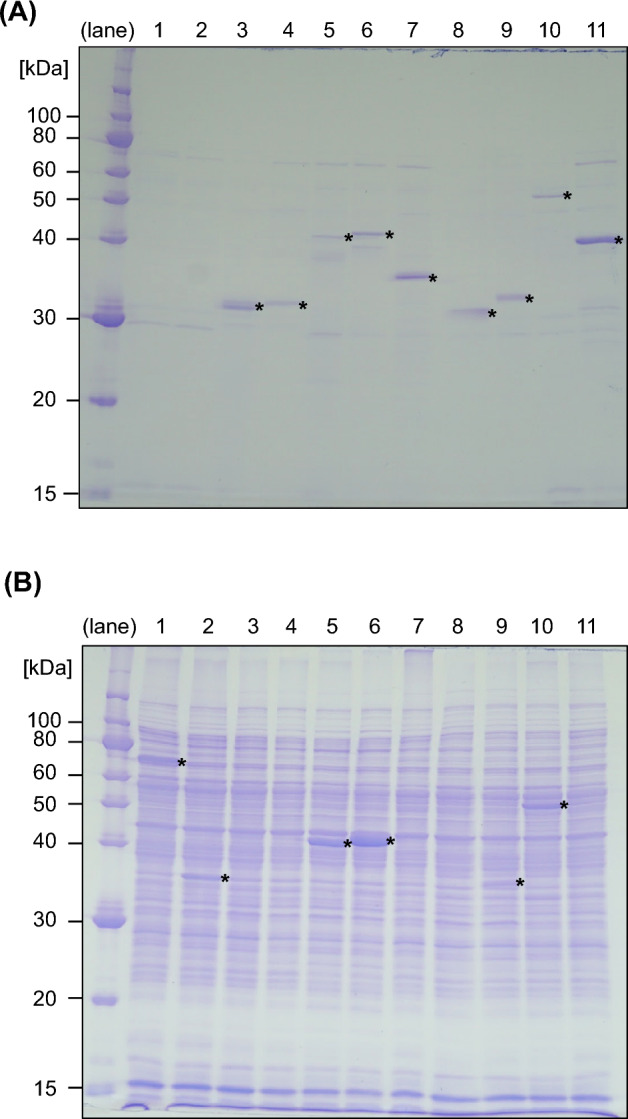
High-level production of secreted enzymes. (**A**) iLiEX-i transformants of *S. griseus* harboring GOIs encoding GUS (lane 1), XylE (lane 2), leucine aminopeptidase (LAP) from *S. griseus* (lane 3) and *S. pratensis* (lane 4), prolyl aminopeptidase from *S. griseus* (lane 5) and *S. lividans* (lane 6), transglutaminase from *S. mobaraensis* (lane 7), small laccase from *S. coelicolor* A3(2) (lane 8) and from *Streptomyces* sp. AGRT-94 (lane 9), catalase-peroxidase of *Streptomyces* sp. No. 565 (lane 10), and xylanase from *Streptomyces* sp. AGRT-94 (lane 11) were grown in shaking culture for 48 h under blue light conditions. Cell-free culture supernatant (10 μL) were separated using SDS-PAGE and stained with CBB. (**B**) The total intracellular proteins of the iLiEX-i transformants were separated using SDS-PAGE and stained with CBB. The asterisk (*) indicates the band corresponding to each enzyme. The original, unprocessed images of the full-length gel are included in Supplementary Fig. S9 and S10.

Enzymes secreted by *Streptomyces* spp. are utilized in the industry, including transglutaminase^8^, leucine aminopeptidase (LAP)^50^, and phospholipase A2^51^. To evaluate the suitability of iLiEX-i for the high-level production of secreted enzymes, we examined nine potentially high-utility enzymes. Transformants carrying each gene were cultured under light conditions and the secreted enzymes in the culture supernatant (10 μL) were separated and detected by SDS-PAGE with CBB staining. We did not concentrate the proteins because we detected protein bands in 10 μL of the culture supernatant in our preliminary experiments. As shown in Fig. 9A and Supplementary Fig. S9, we detected CBB-stained bands near the predicted molecular weights of nine enzymes: LAP from *S. griseus* NBRC 13350 and *S. pratensis* DSM 40990, prolyl aminopeptidase^52^ from *S. griseus* NBRC 13350 and *S. lividans* 66, transglutaminase^8^ from *S. mobaraensis* NBRC 13819, small laccase^53,54^ from *S. coelicolor* A3(2), and *Streptomyces* sp. AGRT-94 is a catalase-peroxidase isolated from *Streptomyces* sp. No. 565 and xylanase^6^ from *Streptomyces* sp. AGRT-94. We also analyzed the cytoplasmic proteins in cell lysates prepared by sonication (Fig. 9B and Supplementary Fig. S10). Protein bands near the predicted molecular weights were detected in the cytoplasmic fractions of prolyl aminopeptidases from *S. griseus* and *S. lividans* 66, a small laccase from *Streptomyces* sp. AGRT-94, and catalase peroxidase from *Streptomyces* sp. No. 565. GUS and XylE, which were used as controls for cytoplasmic proteins, were not secreted. We successfully developed a single-copy and light-inducible system for large-scale production of *Streptomyces* secreted enzymes.

### High-level production of Leucine aminopeptidase from the early growth phase

**Fig. 10:**
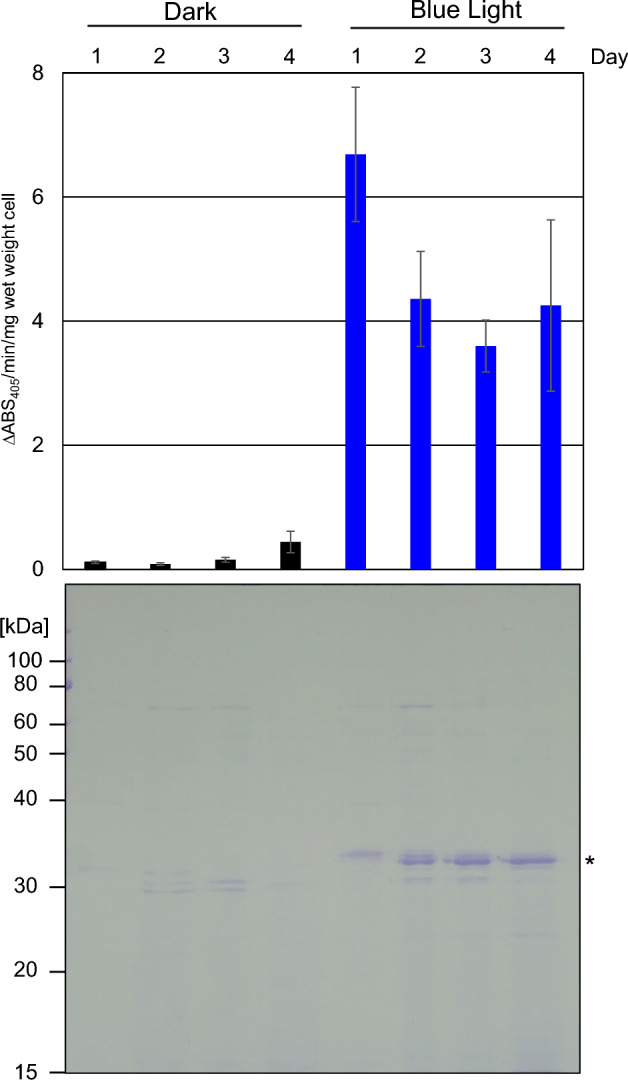
High production of LAP from the early growth phase. The iLiEX-i transformant of *S. griseus* harboring LAP was grown in a shaking culture under dark and blue light conditions. The LAP activity in the culture supernatant was evaluated every 24 h using a chromogenic substrate and calculated as the change in absorbance at 405 nm per minute per milligram of total protein (ΔABS405 nm/min/mg). Data represent the mean ± SD from three independent experiments (*n* = 3). Cell-free culture supernatants (10 μL) prepared every 24 h for four days were separated using SDS-PAGE and stained with CBB (shown at the bottom). Asterisk (*) indicates the band corresponding to LAP. The original unprocessed images of the full-length gel are shown in Supplementary Fig. S11.

LAP is an industrial food enzyme derived from *S. griseus* that reduces bitterness and enhances flavor^50^. The LAP production was evaluated every 24 h using a chromogenic substrate. High activity was detected 24 h after the start of the culture under blue light conditions (Fig. 10 and Supplementary Fig. S11). Analysis of 10 μL of heat-denatured culture supernatant using SDS-PAGE followed by CBB staining to reveal significant protein secretion as early as 24 h after incubation. This suggests that the iLiEX-i system is well-suited for producing secreted enzymes during the early growth phase.

## Discussion

Two genome-integrated iLiEX systems were developed to address the narrow host range of high-copy plasmid pLiEX systems^23^. Of these two types, we demonstrated that iLiEX-i functions across a broad range of *Streptomyces* species with high-level production of beneficial enzymes, thereby overcoming the narrow host range limitation. All components of the iLiEX system are integrated into the genome; therefore, no gene dosage effects are expected. However, by utilizing T7RNAP as a transcriptional amplifier, the prototype iLiEX-i exhibited productivity comparable to that of the plasmid-based system. Furthermore, the addition of an RBS significantly enhanced the functionality of the iLiEX-i system. Serine-type int vectors in *Streptomyces* are less likely to drop out of transformants in the absence of antibiotic selection pressure. In our analysis using *S. griseus*, the dropout frequency of φC31 and φBT1 integration vectors from the genome was 0% after three culture passages. In addition, the transformation efficiency of the int vector by conjugative transfer was extremely high for *Streptomyces* spp. Therefore, the iLiEX-i system offers several advantages, including environmental friendliness, cost and time efficiency, and minimal burden on *Streptomyces* cells.

We demonstrated that T7RNAP functions effectively in *Streptomyces* spp. as a transcriptional amplification device (Figs. 2 and 3). To date, only two examples of T7RNAP used in the study of *Streptomyces* have been reported. Lussier et al.^55^ succeeded in secreting Xylanase in *S. lividans* using genome-integrated *T7RNAP* under a thiostrepton-regulated promoter, and the xylanase gene was expressed from the high-copy plasmid pJV1. In another study, Wei et al.^56^ reported the production of the pigmented antibiotic actinorhodin in *S. coelicolor*, in which T7RNAP was used to enhance the expression of ActII-ORF4, a pathway-specific transcriptional activator, which increased the maximum yield of actinorhodin by 15-fold. Taken together, our results and those of previous studies indicate that T7RNAP is functional in *Streptomyces* spp. However, it remains unclear whether T7RNAP is suitable for transcribing large secondary metabolite biosynthesis enzymes such as type I polyketide synthases (a few kb to tens of kb). We plan to apply iLiEX on secondary metabolite production in future studies.

The iLiEX-i system functions extensively across the bacterial genus *Streptomyces* (Fig. 4). The hosts included *S. coelicolor*, *S. lividans*, *S. albus* J1074, and *S. avermitilis,* which are used to produce valuable substances and enzymes. Why is the one-plasmid-type functional in only a limited number of *Streptomyces* strains, whereas the genome-integrative-type exhibits a broader host range? The mechanism is unknown; however, the following hypothesis is proposed: LitR requires coenzyme B_12_ to function properly, which is the ligand responsible for photoreception and biosynthesized in *Streptomyces*. Therefore, it is expected that a large number of LitR proteins expressed in high copy-number plasmids will function normally only in strains with a high supply of B_12_. In contrast, for genomic single-copy iLiEX system, the required B_12_ supply is expected to be similar to the physiological level in the wild-type strain. *S. avermitilis* likely has a low capacity for intracellular B_12_ synthesis, as exogenous B_12_ is needed for the normal function of LitR. The findings suggest that pLiEX may only function effectively in strains such as *S. griseus*, which has a high ability to produce B_12_.

We successfully improved the functionality of the iLiEX system by addressing two key aspects: (i) improving productivity through RBS optimization (Fig. 5), and (ii) achieving precise control by suppressing leaky expression with LysY or LysS (Fig. 6). RBS_*nitA*_, derived from the nitrile hydratase gene of *Rhodococcus rhodochrous*^18^, was found to be more suitable for protein production. Herai et al. used RBS_*nitA*_ to develop an epsilon-caprolactam-induced expression system that functioned in *Streptomyces* spp^18^. Although the physiological characteristics of *Rhodococcus* and *Streptomyces* are vastly different, they share several features, including genome size, codon usage frequency, and RBS. High leakage under dark conditions is a drawback of the iLiEX system, which was resolved using LysY or LysS. Its use also reduced the level of transcription under light conditions. However, we successfully constructed three types of expression systems with varying output intensities (weak, medium, and strong activity). It is envisioned that stepwise control of the promoter activity will be necessary for future precision fermentation.

We succeeded in shortening the *T7* promoter length, thereby increasing the versatility of the iLiEX-i system (Fig. 7). The wild-type *T7* promoter, derived from the T7 phage and functioning in *S. griseus*, is 89 bp long and costly in terms of oligonucleotide synthesis. By shortening the spacer region, we successfully reduced its length to 44 bp without compromising the transcription. The sequence of the standard *T7* promoter is 5′-TAATACGACTCACTATATA*G*GGAGA-3′, 23 bp long, with base G (underlined) being the transcription start site (+ 1). The spacer region forms part of the 5′ untranslated region (5'-UTR) of mRNA, which may contain regulatory elements that influence ribosome binds and initiate translation at the start codon. The 5’-UTR has not been thoroughly investigated, and its function remains unclear. In future studies, we plan to introduce random mutations in the shortened 44 bp to develop a *T7* promoter that is more active in *Streptomyces* spp. To enhance the versatility of the iLiEX system, we also attempted to combine the reporter and booster elements on a single plasmid. However, we were unable to construct such a plasmid using either pUC19- or p15A-based plasmid, for reasons that remain unclear.

We successfully expressed *sfGFP*, *mScarlet-I*, *rppA*, and *aeBlue* via single-copy integration into the genome (Fig. 8). In our preliminary experiments, the *sfGFP* gene, driven by the strong promoter ermE*p, was introduced into the genome of *S. griseus*. However, no green fluorescence was observed when the transformant was grown on solid agar medium and irradiated with blue light from an LED. Therefore, transcriptional amplification by T7RNAP is essential for visually observing the fluorescence emitted by colonies. In the iLiEX-i system, the copy number of the introduced GOI gene was equivalent to that of the genome, avoiding any artificial increase in gene copy numbers. Therefore, the physiological state of the transformants was considered relatively close to that of the wild-type strain. Another outstanding point is that T7RNAP tended to be transcriptionally amplified, maintaining the transcription fold ratio of the wild-type promoter. Furthermore, even without the addition of selective antibiotics to the culture medium, genome-integrative vectors rarely drop out of the genome, allowing for the analysis of cells in a physiological state similar to that of the wild type with a low burden on the host. Therefore, the iLiEX system is expected to have a wide range of applications, including the development of chemically inducible production systems and chemical sensor strains.

We successfully produced beneficial enzymes, including leucine aminopeptidase, prolyl aminopeptidase, transglutaminase, small laccase, catalase-peroxidase, and xylanase, in large quantities via mass production using iLiEX-i (Figs. 9 and 10). Notably, we have found no reports of recombinant protein production using a single-copy genome-integrative vector where a protein band was detected using SDS-PAGE followed by CBB staining of 10 μL culture supernatant. Furthermore, Leucine aminopeptidase is a suitable reporter enzyme for analyzing gene expression because it is produced in the early stages of culture. However, compared to the high-copy plasmid-based pLiEX system^23^, production in the iLiEX-i system remains low, as exemplified by the small amount of laccase. Therefore, further improvements, such as cascading boosters, are required to enhance the iLiEX-i system to an industrial level.

We successfully developed a single-copy light-inducible system, iLiEX, that is broadly functional in *Streptomyces*. It is expected that this system can be used for the mass production of industrial enzymes when combined with a strong constitutive promoter. In *Streptomyces*, many chemical (sugar and antibiotic) sensors and their cognate promoters have been identified; however, most of them have not been utilized in production systems because they do not function on multicopy plasmids or exhibit weak promoter activity in a single copy. The core system of iLiEX is expected to be widely applied in *Streptomyces* production systems in future studies.

## Methods

### Bacterial strains, plasmids, chemicals, and culture media

*S. griseus* NBRC 13350 (previously known as IFO 13350), *Streptomyces* sp. NBRC 13304 (formerly known as *S. griseus*), *S. avermitilis* MA-4680 (NBRC 14893), and *S. lividans* 66 (NBRC 15675) were obtained from the Institute of Fermentation (Osaka, Japan) and the NITE Biological Resource Center (Chiba, Japan). *S. coelicolor* A3(2) M145, *S. lividans* TK24, and *S. albus* J1074 were obtained from John Innes Center. *E. coli* K-12 strain HST08 (Takara Bio Inc., Shiga, Japan) was used for routine cloning experiments, and HST04 (Takara Bio) carrying pUB307-aphA::Tn7/pTH18cr-539 was used as a helper strain for intergeneric conjugation between *E. coli* and *Streptomyces.* pIJ702, a Streptomyces self-replicating high-copy plasmid harboring the thiostrepton resistance gene (tsr), was used to construct the iLiEX-p system. pLit19, pLit19-XylE, and pLit19-GUS were used as a one-plasmid pLiEX system^23^. pTYM19, provided by H. Onaka, was employed to construct *E. coli–Streptomyces* shuttle plasmid vectors. The kanamycin-resistant *E. coli* plasmid, pK18mobsacB, was used to construct the int vector as a chassis vector. pKU463 and pKU464, provided by H. Ikeda, were used as PCR templates for the φK38-1 and φBT1 integrases.

*Streptomyces* were cultivated in the following media; YMP/sugar medium (per liter: 2 g yeast extract, 2 g meat extract, 4 g Bacto peptone [Becton, Dickinson and Co., Sparks, MD], 5 g NaCl, 2 g MgSO_4_.7H_2_O, and 10 g Glucose or Maltose [Kokusan, Tokyo, Japan], [pH 7.2]), Bennett’s/sugar medium (per liter: 1 g yeast extract [Becton, Dickinson and Co.], 1 g fish extract [Kyokuto, Tokyo, Japan], 2 g NZ amine [FUJIFILM Wako Pure Chemical corp., Osaka, Japan], and 10 g Glucose or Maltose [Kokusan], pH 7.2), Agar (1.5%; Kokusan) was added to each mixtures to prepare the solid media. For transformant selection and plasmid maintenance in *Streptomyces* and *E. coli*, antibiotics were used at the following concentrations: ampicillin (Wako) and kanamycin (Wako) at 50 μg/mL, chloramphenicol at 20 μg/mL, tetracycline at 10 μg/mL, and spectinomycin (Wako) at 60 μg/mL for *E. coli*; thiostrepton (Santa Cruz Biotechnology, DAL, USA) at 20 μg/mL, and kanamycin at 20 μg/mL for *Streptomyces*.

### Light irradiation method during culture

Incubators were equipped with blue LED light irradiation units (TH2-300X300BL; CCS Inc., Kyoto, Japan; λ_max_ = 450 nm) for shaking liquid and solid cultures. Light intensity, expressed as photosynthetic photon flux density (PPFD), was measured using a LI-250 Light Meter (LI-COR Inc., Lincoln, NE, USA).

### Artificially synthesized DNA fragments

The following GOIs or promoter-GOI cassettes were designed with codon usage frequencies optimized for *S. coelicolor* A3(2) and synthesized artificially using Twist Bioscience (SF, USA): *T7RNAP* of T7 phage, *T7* promoter-MCS-*T7* terminator (T7p-MCS), P*hrdB*-*lysS*, P*hrdB*-*lysY*, *aeBlue*, and *mScarlet-I*. *sfGFP*, a codon optimized for *S. venezuelae* ATCC 10712 based on a previous study[57], was synthesized. The DNA nucleotide sequences are listed in Supplementary Table S1.

### Standard DNA manipulations

DNA manipulation of *E. coli* and *Streptomyces* spp. was performed following the protocols described by Sambrook et al.^58^ and Kieser et al.^59^, respectively. Synthetic oligonucleotides (Supplementary Table S2) purchased from Eurofins Genomics K.K. (Tokyo, Japan) were used for vector construction and cloning experiments. All the enzymes were purchased from Takara Bio and New England BioLabs (NEB, Ipswich, MA, USA). Standard PCR was carried out using PrimeSTAR GXL Premix, PrimeSTAR Max Premix, Q5 High-Fidelity 2X Master Mix, or Ex Premier DNA Polymerase. Plasmid and PCR amplicon DNA were purified using the QIAprep Spin Miniprep Kit and the QIAquick Gel Extraction Kit (Qiagen GmbH, Hilden, Germany). All experiments were performed according to the manufacturer’s instructions. DNA sequencing was performed by Eurofins Genomics.

### Transformation methods of *Streptomyces* strains with high-copy-number plasmids and integrative vectors

To transform *S. griseus* NBRC 13350, *S. coelicolor* A3(2), and *S. lividans*, pIJ702-based high-copy-number plasmids were introduced using the protoplast-PEG method^60,61^, and thiostrepton-resistant transformants were selected. Integration vectors were introduced into a conjugation helper strain*, E. coli* HST04/pUB307-*aphA*::Tn7/pTH18cr-539*,* to methylate the *S. griseus* type^62^, or *E. coli* HST04/pUB307-*aphA*::Tn7 for *S. albus* J1074, *Streptomyces* sp. NBRC 13304, *S. avermitilis*, *S. lividans*, and *S. coelicolor* A3(2). Intergeneric conjugation between the *E. coli* helper strain harboring the integration vector and *Streptomyces* strains was performed as previously described^59^.

### Construction of backbone vectors for genome integration

To construct kanamycin-resistance vectors pNU463 and pNU464, a genome-integrative vector retaining φK38-1 integrase and φBT1 integrase, respectively, *int* genes were amplified using primer pair P01/P02 for φK38-1 int and P03/P04 for φBT1 int with pKU463 and pKU464 (both kindly provided by H. Ikeda) serving as templates. The resulting amplicons were introduced into the *Bst*BI site of pK18mob using In-Fusion cloning method (Takara Bio), yielding plasmids pNU463 and pNU464.

pTYM19 (kindly provided by H. Onaka), a thiostrepton-resistant genome-integrative vector, contains two *Nde*I sites upstream and downstream of the φC31 integrase gene [63]. To remove the *Nde*I sites, pTYM19vio (kindly provided by H. Onaka) was digested with *Bam*HI, and the linearized vector was self-ligated to yield pTYM19Δ.

To construct pTYM19Δ-T7p-MCS, the *T7* promoter-MCS-*T7* terminator cassette was amplified by PCR using the primer pair P05/P06 and inserted into the *Eco*RI and *Hin*dIII sites of pTYM19Δ to generate pTYM19Δ-T7p-MCS.

### Construction of genome-integrative booster vectors

To construct the booster int vector, the *litR*-*litS*-*crtE*p operon and the codon-optimized *T7RNAP* gene were amplified by PCR using primer pairs P07/P08 (with pLit19 as the template), and P09/P10 (with synthetic *T7RNAP* DNA as the template), respectively. These two amplicons were inserted between the *Kpn*I and *Hin*dIII sites of pNU464 and pNU463 using In-Fusion, yielding pNU464-litRS-T7RANP and pNU463-litRS-T7RANP, respectively.

To construct *lys*-harboring booster vectors, DNA fragments (Twist Biosciences) containing either P*hrdB*-*lysS* or P*hrdB*-*lysY*, each with a short overlapping sequence for In-Fusion, were inserted into the *Kpn*I site of pKU464-LitRS-T7RNAP, yielding pKU464-LitRS-T7RNAP-lysS and pKU464-LitRS-T7RNAP-lysY.

### Construction of pIJ702-based high-copy reporter plasmids

To construct the pIJ702-based high-copy number plasmid, the *T7* promoter-MCS-*T7* terminator cassette was amplified by PCR using synthetic DNA as a template with the primer pair P11/P12. A cassette was inserted between *Kpn*I and *Mfe*I sites of pLit19ΔNdeI to yielding pIJ702-T7p-MCS-T7ter. GOI were amplified using the primer pair P13/P14 for *xylE* and P15/ P16 for *gus* and inserted between *Nde*I and *Hin*dIII sites of pIJ702-T7p-MCS-T7ter with appropriate restriction enzymes and T4 Ligase (Takara Bio), yielding pIJ702-T7p-xylE and pIJ702-T7p-gus, respectively. The constructed plasmids were introduced into *S. griseus* via the protoplast-PEG method, and thiostrepton-resistant transformants were selected. Plasmids were purified from the transformants as described in our previous study [23].

### Construction of reporter int vectors

GOIs were amplified by PCR using the following primer pairs and cloned into the *Nde*I/*Bam*HI or *Nde*I/*Hin*dIII sites of pTYM19-T7p-MCS; P17/P18 for *xylE*, P19/P20 for *gus*, and P21/P22 for *rppA* (*SGR_6620*) of *S. griseus* NBRC 13350, P23/P24 for LAP (*SGR_5809*) of *S. griseus* NBRC 13350, P25/P26 for LAP (*Sfla_5137*) of *S. pratensis* DSM 40990, P27/P28 for prolyl aminopeptidase (*SGR_0506*) from *S. griseus* NBRC 13350, P29/P30 for prolyl aminopeptidase (*SLV_33905*) from *S. lividans* 66, P31/P32 for transglutaminase from *S. mobaraensis* NBRC 13819 (Supplementary Table S1), P33/P34 for small laccase (*SCO6712*) from *S. coelicolor* A3(2), P35/P36 for small laccase from *Streptomyces* sp. AGRT-94 (Supplementary Table S1), and P37/P38 for the catalase-peroxidase of *Streptomyces* sp. No. 565 (Supplementary Table S1) and P39/P40 for xylanase from *Streptomyces* sp. AGRT-94 cells (Supplementary Table S1).

### Construction of pNU464-LitRS-crtEp-GUS and pNU464-ermE*p-GUS

To construct the *gus* reporter vector, a DNA fragment containing *gus* was amplified using the primer pair P41/P42 with pLit19-GUS^23^ as a template. The resulting amplicon was inserted between the *Pst*I and *Hin*dIII sites of pNU464, yielding pNU464-gus. To construct promoter variants, DNA fragments containing *litR*-*litS*-*crtE*p and ermE*p were amplified using primer pair P43/P44 with *litR*-*litS*-*crtE*p using pLit19 as a template, and P45/P46 for ermE*p using the PCR template pBPSA1^64^. These fragments were independently inserted between the *Pst*I and *Nde*I sites of pKU464GUS to generate pNU464-LitRS-*crtEp*-GUS and pNU464-ermE*p-GUS, respectively.

### Construction of pNU464-LitRS-T7RNAP with modified RBS and start codon

The following primer pairs were used to amplify each pair of RBS/start codons: P47/P48 for RBS_*nitA*_/ATG, P49/P50 for RBS_*nitA*_/GTG, P51/P52 for RBS_*nitA*_/TTG, P53/P54 for RBS_*w52*_/ATG, P55/ P56 for RBS_*sav2794*_/ATG, using pLit19 as the PCR template. The amplicons were inserted between the *Kpn*I and *Nde*I sites of pNU464-RS-T7RNAPst using In-Fusion.

### Trimming of *T7* promoter

To shorten the spacer region of the wild-type *T7* promoter (43 bp), PCR forward primers were used to create fragments of 13 bp (P57), 11 bp (P58), 9 bp (P59), 7 bp (P60), and 5 bp (P61). The commonly used PCR reverse primer is P62. Each PCR amplicon, retaining the trimmed *T7* promoter and sfGFP, was introduced via In-Fusion cloning into pTYM19 between the *Eco*RI and *Hin*dIII sites.

### Analysis of the enzymatic activities for XylE, GUS, and LAP

Transformants were cultured in 100-mL baffled Erlenmeyer flasks containing 20 mL of either YMP-glucose medium (for *S. griseus*) or TSB medium (for *S. albus* J1074, *Streptomyces* sp. NBRC 13304, *S. avermitilis*, *S. lividans*, and *S. coelicolor* A3(2)), and incubated at 28 °C for 48 h on a rotary shaker (135 rpm) under both dark and blue light (λ_max_ = 450 nm, PPFD: 3 mol s^−1^ m^−2^) conditions. According to our previous study^23^, the enzymatic activities of XylE and GUS were measured using catechol and *p*-nitrophenyl-β-D-glucuronide as chromogenic substrates, respectively^34,65^. For the LAP assay, *p*-nitrophenol formation was monitored in a 200 µL reaction mixture containing 100 µL of Basal buffer (1 mM Tris–HCl (pH 8.0), 1 mM CaCl_2_, and 0.4 mg/mL L-Leucyl-*p*-nitroanilide Hydrochloride (pNA, Wako) as a chromogenic substrate)[66] and 2 or 10 µL of culture supernatant. Absorbance at 405 nm were measured every 30 s for 11 min at 37 °C using Multiskan GO or Multiskan SkyHigh (Thermo Fisher Scientific, Rockford, IL, USA). LAP activity was calculated as the change in absorbance at 405 nm/min per mg of wet cell weight as the protein present in the culture medium was low and the protein concentration could not be accurately measured. For the XylE and GUS assays, the protein concentrations in the cell lysates were measured using a NanoDrop Lite Plus spectrophotometer (Thermo Fisher Scientific). The wet cell weight was used to assess *Streptomyces* cell growth. A 1.0 mL culture broth was harvested by centrifugation (16,230 × g for 5 min), and the pellets were washed with 40% glycerol solution to prevent pellet collapse. The washed cells were harvested by centrifugation, and the wet cell weight was calculated by subtracting the previously measured weight of the 1.5 mL tube using a precision electronic balance.

As in our previous study^23^, proteins produced in the cytoplasm or culture supernatant were heat-denatured, separated by sodium dodecyl sulfate–polyacrylamide gel electrophoresis (SDS-PAGE), and stained with Coomassie Brilliant Blue R-250 (CBB). For molecular weight estimation, the XL-Ladder Broad (Apro Science Group, Pharma Foods International Co., Ltd., Tokushima, Japan) was used as a molecular weight marker.

### Significance statement

We previously developed a light-inducible gene expression (LiEX) system based on a high copy-number plasmid in *Streptomyces griseus*. However, its host range is limited, as it functions in only a few species of *Streptomyces*. In this study, we achieved high-level light-inducible expression from a single copy integrated into the genome using viral T7 RNA polymerase. This single-copy system broadens the applicability of LiEX to a wide range of *Streptomyces* species.

## Supplementary Information

Supplementary Information.

## Data Availability

The datasets generated in this study are available from the corresponding author upon request.
