# Supplementary material for: Single copy optogenetic system for Streptomyces
Source: Sci Rep. 2025 Dec 15;15:43857. doi: 10.1038/s41598-025-27850-9 (PMC12705666; doi:10.1038/s41598-025-27850-9)
Supplement: Supplementary file 1 — Supplementary Information. [file 41598_2025_27850_MOESM1_ESM.pdf]

Supplementary Information

Single Copy Optogenetic System for *Streptomyces*

Airi Watanabe, Ryuta Noya, Rio Yamada, and Hideaki Takano*

Life Science Research Center, College of Bioresource Sciences, Nihon University

*Corresponding author.
Hideaki Takano
Life Science Research Center, College of Bioresource Sciences, Nihon University
1866 Kameino, Fujisawa 252-0880 Japan
Tel; +81-466-84-3936, Fax; +81-466-84-3935
E-mail; takano.hideaki@nihon-u.ac.jp

Supplementary Table S1 Nucleotide sequences of DNAs used in this study

Name	Nucleotide sequences (5'-3')
T7 RNA polymerase<br><br>(Codon optimized for *S. coelicolor* A3(2))	ATGAACACCATCAACATCGCCAAGAACGACTTCTCCGACATCGAGCTCGCCGCGATCCCCTTCAACACGCTCGC<br>CGACCACTACGGCGAACGCCTCGCCCGGGAGCAGCTGGCGCTCGAACACGAATCCTACGAAATGGGCGAGGCCC<br>GGTTCCGGAAGATGTTCGAACGGCAGCTGAAGGCCGGTGAAGTCGCCGACAACGCGGCGGCGAAGCCCCTGATC<br>ACGACGCTGCTGCCCAAGATGATCGCCCGGATCAACGACTGGTTCGAAGAGGTCAAGGCCAAGCGCGGGAAGCG<br>GCCCACCGCGTTCCAGTTCCTGCAGGAGATCAAGCCCGAGGCGGTCGCCTACATCACGATCAAGACGACCCTCG<br>CGTGTCTGACGTCGGCCGACAACACGACGGTCCAGGCCGTCGCGTCCGCCATCGGCCGCGCGATCGAAGACGAA<br>GCCCGGTTCGGCCGCATCCGCGACCTCGAAGCCAAGCACTTCAAGAAGAACGTCGAAGAGCAGCTGAACAAGCG<br>GGTCGGCCACGTGTACAAGAAGGCCTTCATGCAGGTGGTGGAAGCCGACATGCTGTCGAAGGGCCTGCTGGGCG<br>GGGAAGCCTGGTCCTCGTGGCACAAGGAAGACTCCATCCACGTGGGTGTCCGGTGTATCGAAATGCTGATCGAA<br>AGCACGGGCATGGTCTCGCTCCACCGGCAGAACGCCGGAGTGGTCGGCCAGGACTCGGAAACGATCGAGCTGGC<br>GCCGGAATACGCGGAAGCCATCGCCACGCGGGCCGGCGCCCTCGCGGGAATCAGCCCCATGTTCCAGCCGTGTG<br>TGGTGCCGCCCAAGCCCTGGACCGGGATCACCGGCGGCGGGTACTGGGCCAACGGCCGCCGGCCGCTCGCCCTC<br>GTCCGGACCCACTCCAAGAAGGCGCTCATGCGGTACGAGGACGTGTACATGCCCGAAGTCTACAAGGCCATCAA<br>CATCGCCCAGAACACGGCGTGGAAGATCAACAAGAAGGTGCTGGCCGTGGCGAACGTGATCACGAAGTGGAAGC<br>ACTGCCCCGTGGAAGACATCCCCGCCATCGAACGCGAGGAGCTGCCCATGAAGCCCGAGGACATCGACATGAAC<br>CCCGAAGCGCTGACGGCCTGGAAGCGGGCCGCGGCCGCGGTCTACCGGAAGGACAAGGCCCGGAAGTCCCGGCG<br>CATCTCGCTGGAATTCATGCTCGAACAGGCGAACAAGTTCGCCAACCACAAGGCGATCTGGTTCCCGTACAACA<br>TGGACTGGCGGGGCCGGGTGTACGCCGTCAGCATGTTCAACCCCCAGGGCAACGACATGACGAAGGGGCTCCTC<br>ACCCTCGCCAAGGGGAAGCCCATCGGCAAGGAAGGCTACTACTGGCTCAAGATCCACGGAGCGAACTGCGCCGG<br>CGTGGACAAGGTGCCCTTCCCCGAACGGATCAAGTTCATCGAAGAGAACCACGAAAACATCATGGCCTGTGCGA<br>AGTCCCCGCTCGAAAACACCTGGTGGGCGGAACAGGACTCCCCCTTCTGTTTCCTGGCCTTCTGTTTCGAATAC<br>GCCGGCGTGCAGCACCACGGTCTCTCCTACAACTGTAGCCTGCCCCTCGCCTTCGACGGCTCGTGTTCCGGCAT<br>CCAGCACTTCTCGGCCATGCTGCGGGACGAAGTGGGCGGCCGGGCCGTGAACCTGCTGCCGTCGGAGACGGTGC<br>AGGACATCTACGGCATCGTGGCCAAGAAGGTGAACGAAATCCTGCAGGCGGACGCCATCAACGGCACGGACAAC<br>GAGGTGGTCACGGTCACGGACGAAAACACCGGCGAAATCTCGGAAAAGGTGAAGCTCGGGACCAAGGCCCTCGC<br>CGGCCAGTGGCTCGCCTACGGAGTGACCCGGTCGGTCACCAAGCGCAGCGTGATGACCCTCGCCTACGGAAGCA<br>AGGAATTCGGATTCCGCCAGCAGGTCCTCGAAGACACGATCCAGCCCGCCATCGACTCGGGGAAGGGCCTCATG<br>TTCACCCAGCCCAACCAGGCCGCCGGTTACATGGCCAAGCTCATCTGGGAGAGCGTCTCCGTCACCGTCGTCGC<br>CGCCGTGGAAGCCATGAACTGGCTGAAGTCCGCGGCCAAGCTCCTCGCCGCGGAAGTGAAGGACAAGAAGACGG<br>GCGAAATCCTGCGGAAGCGCTGTGCGGTCCACTGGGTGACGCCCGACGGCTTCCCCGTCTGGCAGGAGTACAAG<br>AAGCCCATCCAGACCCGGCTGAACCTCATGTTCCTGGGCCAGTTCCGGCTCCAGCCGACGATCAACACGAACAA<br>GGACTCGGAAATCGACGCCCACAAGCAGGAATCCGGAATCGCCCCCAACTTCGTGCACTCGCAGGACGGCTCCC<br>ACCTGCGGAAGACCGTCGTCTGGGCCCACGAAAAGTACGGGATCGAGAGCTTCGCCCTCATCCACGACTCGTTC<br>GGAACGATCCCCGCCGACGCGGCCAACCTCTTCAAGGCGGTCCGGGAGACCATGGTGGACACGTACGAATCCTG<br>CGACGTCCTCGCCGACTTCTACGACCAGTTCGCCGACCAGCTCCACGAAAGCCAGCTCGACAAGATGCCGGCCC<br>TGCCCGCGAAGGGCAACCTCAACCTGCGCGACATCCTGGAAAGCGACTTCGCCTTCGCCTGA
T7 promoter-RBS-6xHis-**T7 terminator**	GGTACCAGGATCTCGATCCCGCGAAATTAATACGACTCACTATAGGGAGACCACAACGGTTTCCCTCTAGAAAT<br>AATTTTGTTTAACTTTAAGAAGGAGATATACATATGGGCCATCACCATCACCATCACGCCATGGGAATTCTACG<br>TAGCGGCCGCGGATCCAAGCTTAGGTCTCTCGAGCATCACCATCACCATCACTGAGGCTGCTAACAAAGCCCGA<br>AAGGAAGCTGAGTTGGCTGCTGCCACCGCTGAGCAATAAC**TAGCATAACCCCTTGGGGCCTCTAAACGGGTCTT**<br>**GAGGGGTTTTTTG**CTGAAAGGAGAGATCT
**P*hrdB*-*lysS***<br><br>(Codon optimized for *S. coelicolor* A3(2))<br><br>(Underlined: overlap sequence for seamless cloning)	CTAGAGGATCCCCGGATAATTGTGGGGGGATGGCACCTTCGGCCCATTTCGTCACGTATGAGGTGTGACTCGGG<br>CCACGCGGATTGGCCGTAACACTCCTCGAAGCAGCGCGATGACTTAAGAGGTGAGCGTCGCGGAAGGAATACAG<br>CAGCCCTCGGGTGTGCTGTCCAGTTCCGAGGCCAAACCCGCGCCGTCGGCGACATCCCCAGTCGACGGTCGTCG<br>GTTCCAGCCCTCTCCAGGGCGGGGCCGGAAGCCGTTTCCATCGTTCCGAGAGGTTGCATATGGCCCGCGTCCAG<br>TTCAAGCAGCGCGAGTCCACCGACGCCATCTTCGTCCACTGCTCGGCCACCAAGCCCTCCCAGAACGTCGGCGT<br>GCGCGAGATCCGCCAGTGGCACAAGGAGCAGGGCTGGCTGGACGTGGGGTACCACTTCATCATCAAGCGGGACG<br>GCACCGTCGAAGCCGGTCGCGACGAGATGGCGGTCGGCTCGCACGCCAAGGGCTACAACCACAACTCCATCGGC<br>GTCTGCCTGGTCGGCGGGATCGACGACAAGGGCAAGTTCGACGCGAACTTCACGCCGGCCCAGATGCAGAGCCT<br>CCGGTCGCTGCTGGTCACGCTGCTCGCGAAGTACGAAGGCGCCGTGCTCCGCGCCCACCACGAGGTGGCGCCGA

	AGGCGTGCCCCTCCTTCGACCTGAAGCGGTGGTGGGAGAAGAACGAGCTGGTGACCAGCGACCGCGGCTGACGA<br>GCTCGAATTCAC
PhrdB-lysY<br><br>(Codon optimized for *S. coelicolor* A3(2))<br><br>(Underlined: overlap sequence for seamless cloning)	CTAGAGGATCCCCGGATAATTGTGGGGGGATGGCACCTTCGGCCCATTTCGTCACGTATGAGGTGTGACTCGGG<br>CCACGCGGATTGGCCGTAACACTCCTCGAAGCAGCGCGATGACTTAAGAGGTGAGCGTCGCGGAAGGAATACAG<br>CAGCCCTCGGGTGTGCTGTCCAGTTCCGAGGCCAAACCCGCGCCGTCGGCGACATCCCCAGTCGACGGTCGTCG<br>GTTCCAGCCCTCTCCAGGGCGGGGCCGGAAGCCGTTTCCATCGTTCCGAGAGGTTGCATATGGCGCGCGTCCAG<br>TTCAAGCAGCGCGAGAGCACCGACGCCATCTTCGTGCACTGCTCGGCCACCAAGCCCTCGCAGAACGTCGGCGT<br>CCGGGAGATCCGGCAGTGGCACAAGGAGCAGGGCTGGCTGGACGTCGGGTACCACTTCATCATCAAGCGCGACG<br>GGACCGTCGAAGCGGGTCGCGACGAGATGGCCGTGGGCTCCCACGCCAAGGGCTACAACCACAACTCCATCGGC<br>GTCTGCCTGGTGGGCGGCATCGACGACAAGGGCAAGTTCGACGCGAACTTCACGCCCGCCCAGATGCAGTCCCT<br>CCGCTCGCTGCTGGTGACCCTGCTCGCGAAGTACGAAGGCGCCGTGCTCCGGGCCCACCACGAGGTCGCGCCGT<br>ACGCCTGCCCGAGCTTCGACCTGAAGCGCTGGTGGGAGAAGAACGAGCTGGTCACGTCCGACCGCGGCTGACGA<br>GCTCGAATTCAC
aeBlue<br><br>(Codon optimized for *S. coelicolor* A3(2))<br><br>(Underlined: overlap sequence for seamless cloning)	AAGGAGATATACATATGGCGAGCCTCGTGAAGAAGGACATGTGTATCAAGATGACCATGGAAGGTACCGTGAAC<br>GGCCACCACTTCAAGTGCGTCGGCGAGGGAGAGGGTAAGCCGTTCGAGGGTACGCAGGTCGAGAAGATCCGGAT<br>CACCGAGGGCGGCCCGCTGCCGTTCGCCTACGACATCCTCGCGCCCTGTTGTATGTACGGGTCCAAGACGTTCA<br>TCAAGCACGTCTCCGGCATCCCCGACTACTTCAAGGAATCCTTCCCCGAAGGGTTCACGTGGGAGCGGACGCAG<br>ATCTTCGAAGACGGCGGTTACCTGACGATCCACCAGGACACCTCGCTCCAGGGCAACAACTTCATCTTCAAGGT<br>GAACGTGATCGGCGCGAACTTCCCCGCGAACGGACCGGTCATGCAGAAGAAGACGGCCGGCTGGGAGCCCTGTG<br>TCGAAATGCTGTACCCCCGCGACGGAGTGCTCTGCGGGCAGTCGCTCATGGCGCTCAAGTGTACGGACGGAAAC<br>CACCTCACCTCGCACCTGCGGACGACGTACCGCTCCCGGAAGCCGTCGAACGCGGTGAACATGCCCGAGTTCCA<br>CTTCGGCGACCACCGGATCGAAATCCTCAAGGCCGAGCAGGGCAAGTTCTACGAGCAGTACGAATCGGCCGTCG<br>CGCGCTACTGCGAAGCCGCGCCGTCCAAGCTGGGCCACCACTGAAAGCTTAGGTCTCTCG
sfGFP<br><br>(Codon optimized for *S. venezuelae* ATCC 10712)<br><br>(Underlined: overlap sequence for seamless cloning)	AAGGAGATATACATATGTCCAAGGGCGAGGAGCTGTTCACCGGCGTCGTCCCGATCCTGGTCGAGCTGGACGGC<br>GACGTGAACGGCCACAAGTTCTCCGTCCGCGGCGAGGGCGAGGGCGACGCCACCAACGGCAAGCTGACCCTGAA<br>GTTCATCTGCACCACCGGCAAGCTCCCGGTCCCGTGGCCGACCCTGGTCACCACCCTGACCTACGGCGTCCAGT<br>GCTTCTCCCGCTACCCGGACCACATGAAGCGCCACGACTTCTTCAAGTCCGCCATGCCCGAGGGCTACGTCCAG<br>GAGCGGACCATCTCCTTCAAGGACGACGGCACCTACAAGACCCGCGCCGAGGTCAAGTTCGAGGGCGACACCCT<br>GGTCAACCGCATCGAGCTGAAGGGCATCGACTTCAAGGAGGACGGCAACATCCTGGGCCACAAGCTCGAGTACA<br>ACTTCAACTCCCACAACGTCTACATCACCGCCGACAAGCAGAAGAACGGCATCAAGGCCAACTTCAAGATCCGC<br>CACAACGTCGAGGACGGCAGCGTCCAGCTGGCCGACCACTACCAGCAGAACACCCCGATCGGCGACGGCCCGGT<br>CCTGCTGCCGGACAACCACTACCTGTCCACCCAGTCCGTCCTGTCCAAGGACCCGAACGAGAAGCGCGACCACA<br>TGGTCCTGCTCGAGTTCGTCACCGCCGCCGGCATCACCCACGGCATGGACGAGCTGTACAAGTGAAAGCTTAGG<br>TCTCTCG
mScarlet-I<br><br>(Codon optimized for *S. coelicolor* A3(2))<br><br>(Underlined: overlap sequence for seamless cloning)	AAGGAGATATACATATGGTGTCCAAGGGCGAGGCCGTCATCAAGGAGTTCATGCGCTTCAAGGTCCACATGGAG<br>GGCTCCATGAACGGCCACGAGTTCGAGATCGAGGGCGAGGGCGAGGGCCGCCCGTACGAGGGCACCCAGACCGC<br>CAAGCTGAAGGTCACCAAGGGCGGCCCGCTGCCGTTCTCCTGGGACATCCTGTCCCCGCAGTTCATGTACGGCT<br>CCCGCGCCTTCATCAAGCACCCGGCCGACATCCCGGACTACTACAAGCAGTCCTTCCCGGAGGGCTTCAAGTGG<br>GAGCGCGTCATGAACTTCGAGGACGGCGGCGCCGTCACCGTCACCCAGGACACCTCCCTGGAGGACGGCACCCT<br>GATCTACAAGGTCAAGCTGCGCGGCACCAACTTCCCGCCGGACGGCCCGGTCATGCAGAAGAAGACCATGGGCT<br>GGGAGGCCTCCACCGAGCGCCTGTACCCGGAGGACGGCGTCCTGAAGGGCGACATCAAGATGGCCCTGCGCCTG<br>AAGGACGGCGGCCGCTACCTGGCCGACTTCAAGACCACCTACAAGGCCAAGAAGCCGGTCCAGATGCCGGGCGC<br>CTACAACGTCGACCGCAAGCTGGACATCACCTCCCACAACGAGGACTACACCGTCGTCGAGCAGTACGAGCGCT<br>CCGAGGGCCGCCACTCCACCGGCGGCATGGACGAGCTGTACAAGTGAAAGCTTAGGTCTCTCG
transglutaminase<br><br>*S. mobaraensis* NBRC 13819<br><br>(Sec-signal peptide sequence shown by red letters)	ATGCGCATACGCCGGAGAGCTCTCGTCTTCGCCACTATGAGTGCGGTGTTATGCACCGCCGGATTCATGCCGTC<br>GGCCGGCGAGGCCGCCGCCGACAATGGCGCGGGGGAAGAGACGAAGTCCTACGCCGAAACCTACCGCCTCACGG<br>CGGATGACGTCGCGAACATCAACGCGCTCAACGAAAGCGCTCCGGCCGCTTCGAGCGCCGGCCCGTCGTTCCGG<br>GCCCCCGACTCCGACGACAGGGTCACCCCTCCCGCCGAGCCGCTCGACAGGATGCCCGACCCGTACCGTCCCTC<br>GTACGGCAGGGCCGAGACGGTCGTCAACAACTACATACGCAAGTGGCAGCAGGTCTACAGCCACCGCGACGGCA<br>GGAAGCAGCAGATGACCGAGGAGCAGCGGGAGTGGCTGTCCTACGGCTGCGTCGGTGTCACCTGGGTCAATTCG<br>GGTCAGTACCCGACGAACAGACTGGCCTTCGCGTCCTTCGACGAGGACAGGTTCAAGAACGAGCTGAAGAACGG<br>CAGGCCCCGGTCCGGCGAGACGCGGGCGGAGTTCGAGGGCCGCGTCGCGAAGGAGAGCTTCGACGAGGAGAAGG<br>GCTTCCAGCGGGCGCGTGAGGTGGCGTCCGTCATGAACAGGGCCCTGGAGAACGCCCACGACGAGAGCGCTTAC<br>CTCGACAACCTCAAGAAGGAACTGGCGAACGGCAACGACGCCCTGCGCAACGAGGACGCCCGTTCCCCGTTCTA<br>CTCGGCGCTGCGGAACACGCCGTCCTTCAAGGAGCGGAACGGAGGCAATCACGACCCGTCCAGGATGAAGGCCG<br>TCATCTACTCGAAGCACTTCTGGAGCGGCCAGGACCGGTCGAGTTCGGCCGACAAGAGGAAGTACGGCGACCCG

	GACGCCTTCCGCCCCGCCCCGGGCACCGGCCTGGTCGACATGTCGAGGGACAGGAACATTCCGCGCAGCCCCAC<br>CAGCCCCGGTGAGGGATTCGTCAATTTCGACTACGGCTGGTTCGGCGCCCAGACGGAAGCGGACGCCGACAAGA<br>CCGTCTGGACCCACGGAAATCACTATCACGCGCCCAATGGCAGCCTGGGTGCCATGCATGTCTACGAGAGCAAG<br>TTCCGCAACTGGTCCGAGGGTTACTCGGACTTCGACCGCGGAGCCTATGTGATCACCTTCATCCCCAAGAGCTG<br>GAACACCGCCCCCGACAAGGTAAAGCAGGGCTGGCCGTGA
small laccase<br><br>*Streptomyces* sp. AGR T-94<br><br>(Tat-signal peptide sequence shown by red letters)	ATGGACAGACGCGGGTTCAACCGGCGGGTACTGCTCGGCGGGGCGGCGCTGACGACGACATCGTTGTCGCTCGC<br>GCCCCCGGCGAACAGTGCCGTCCAGGCGGCGCGCACGGCGCCCGCGGGCGGCGAGGTCAGGCACATCAGGATGT<br>ACGCCGAGAAGCTCGCCGACGGTCAGATGGGCTACGGCTTCGAGAAGGGGAAGGCGACGATCCCCGGCCCGCTG<br>ATCGAGCTCAACGAGGGCGACACCCTGCACATCGAGTTCGAGAACACCATGGACGTGCCCGTCAGCCTCCATGT<br>GCACGGCCTGGACTACGAGATCACCAGCGACGGCACCAGGCTGAACAAGAGCGACGTCGAGCCCGGCGGCACCC<br>GCACCTACACCTGGCGCACCCACACCCCCGGCCGCCGCAAGGACGGCACCTGGCGGTCGGGCAGCGCGGGTTAC<br>TGGCACTACCACGACCATGTCGTCGGCACCGAACACGGAACGGGCGGTATCCGCAAGGGACTCTACGGGCCGGT<br>GATCGTACGGCGCGCGGGCGATGTGCTGCCGGACAGGACGTACACGATCGTCTTCAACGACATGCTGATCAACA<br>ACAGGCCGGCGCACTCCGGGCCCGACTTCGAGGCCACGGTGGGGGAGCGCGTCGAGTTCGTGATGATCACGCAC<br>GGCGAGTATTACCACACCTTCCACCTGCACGGTCACCGCTGGGCGGACAACCGCACCGGCATGCTCACCGGCCC<br>CGACGACCCCACCCAGGTCATCGACAACAAGATCGTGGGCCCGGCGGACTCCTTCGGCTTCCAGGTCATCGCGG<br>GGGAGGGCGTCGGCGCGGGCGCGTGGATGTACCACTGCCATGTCCAGAGCCACTCCGACATGGGGATGGTGGGG<br>CTGTTCCTGGTGAAGAAGAAGGACGGCACCATCCCCGGGTACGAGCCGCACGACCCGCACGACCCGCACGAGCA<br>CTGA
catalase-peroxidase<br><br>*Streptomyces* sp. No. 565<br><br>(signal peptide sequence is unknown)	GTGACTCAGGGACCGCTTACTACGGAAGCCGGTGCTCCGGTAGCCGACAACCAGAACAGCGAGACCGCGGGCGT<br>CGGCGGCCCCGTGCTCGTCCAGGACCAGCTCCTCCTCGAGAAGCTGGCCCACTTCAACCGTGAGCGCATCCCGG<br>AGCGTGTCGTGCACGCCCGTGGCGCCGGCGCCTACGGCACCTTCACGGTCACCGCCGATGTCACCAAGTACACG<br>CGCGCCGCCTTCCTCTCCGAGGTCGGCAAGGAGACGGAGACCTTCCTCCGCTTCTCGACCGTGGCGGGCAACCT<br>GGGTGCGGCGGACGCCGTCCGTGACCCGCGCGGTTTCGCGCTGAAGTTCTACACCGAAGAGGGCAACTACGACC<br>TCGTCGGCAACAACACCCCGGTGTTCTTCATCAGGGACGCCATCAAGTTCCCCGACTTCATCCACACCCAGAAG<br>CGCGACCCGTACACGGGCTCGCAGGAGGCGGACAACGTCTGGGACTTCTGGAGCCTGTCGCCCGAGTCGACCCA<br>CCAGGTGACCTGGCTGCACGGTGACCGCGGCATCCCGGCCTCCTACCGGCACATGGACGGCTTCGGCTCGCACA<br>CGTACCAGTGGAACAACGAGGCCGGCGAGGCCTTCTGGGTGAAGTACCACTTCAAGACGGACCAGGGCATCAAG<br>AACCTGACCGCCGAGGAGGCCGAGGTCCTCGCGGGCAAGGACCCCGACTCCCATCAGCGCGACCTGCGTGAGGC<br>CATCGAGCGCGGCGACTTCCCGTCCTGGACGGTCGGCGTGCAGATCATGCCCGTCGCCGAGGCCGCCAACTACC<br>GCTTCAACCCGTTCGACCTGACCAAGGTGTGGCCGCACGCGGACTACCCGATCGTCTGGTTCGGCAAGCTGGAG<br>CTCAACCGCAACCCGGAGAACATCTTCGCCGAGGTCGAGCAGTCGATCTTCTCCCCGGCGCACTTCGTGCCCGG<br>CATCGGCCCGTCCCCGGACAAGATGCTCCAGGGCCGTCTCTTCGCCTACGGCGACGCCCACCGCTACCGTGTCG<br>GCATCAACGCCGACCACCTGCCGGTGAACCGCCCGCACGCCACCGAGGCGCGCACCCACTCCCGTGACGGCTAC<br>CTGTACGACGGCCGCCACAAGGGTGCGAAGAACTACGAGCCGAACAGCTTCGGCGGCCCGTTCCAGACGGACAG<br>GGCCCTGTGGCAGCCGGTCGCGGTCTCCGGTGTCACCGGTGACCACGAGACCCCGCGGCACGCCGAGGACAACG<br>ACTTCGTCCAGGCGGGCAACCTCTACCGCCTGATGACGGAGGAGGAGAAGGAGCGCCTGGTCAACAACCTGGCC<br>GGTGGCATCTCCCAGGTCTCGCGCGAGGACATCATCGAGCGCGCGATCAACAACTTCCGTCAGGCCGACGGTGA<br>CTTCGGCAAGCGGCTGGAGGCCGCGGTCCAGGCCCTGCGCGGCTGA
xylanase<br><br>*Streptomyces* sp. AGR T-94<br><br>(Sec-signal peptide sequence shown by red letters)	ATGAAGAACTCACTCGCACGGTTACGACTACTCATCAGCGGCGCCTGTGCCGTGCTGCTGGTCGCGTTCGGCGG<br>GGGCAGTGCGCACGCCGACACCGTGGTCACCACGAATCAGACGGGGACCGACAACGGCTACTACTACTCGTTCT<br>GGACGGACGCCCAGGGCACCGTCTCCATGAACCTCGGCTCCGGCGGGAACTACAGCACGCAGTGGAGGAACACC<br>GGTAACTTCGTCGCCGGCAAGGGCTGGGCCAACGGGTCGCGGCGGACCGTGACCTACTCCGGCACCTTCAATCC<br>GTCCGGCAACGCCTATCTCGCGCTGTACGGATGGACCGCCAACCCGCTCGTCGAGTACTACATCGTCGACAACT<br>GGGGCACCTACCGGCCCACCGGCACCTTCAAGGGCACGGTGACGACCGACGGCGGTACGTACGACATCTACAAG<br>ACGACCCGGTACAACGCCCCGTCCGTGGAAGGCACCAAGACCTTCGACCAGTACTGGAGCGTGCGGCAGTCCAA<br>GCGGACCGGGGGGACCATCACCACCGGCAACCACTTCGACGCCTGGGCCCGCGCCGGCATGCCCCTCGGCAGCT<br>TCAACTACTACATGATCATGGCGACCGAGGGTTACCAGAGCAGCGGCAGCTCCAACATCACCGTCGGCGGCACC<br>GGTGGTGGAGGCGGGGGCGGCGGAGGCGGCGGTGGCGGGTGCAGCGCCACGCTCTCCGCCGGGCAGCAGTGGAG<br>CGACCGGTACAACCTCAACGTCTCGGTCTCCGGCGCCGGCAACTGGACCGTCACCATGAACGTGCCGAGCCCCG<br>CGAAGATCCTCTCCACCTGGAACGTCAGCGCCAGTTACCCCAGTTCACAGGTCCTCACCGCCAAACCCAACGGC<br>AGCGGCAACAACTGGGGCGTCACTATCCAGCACAACGGCAACTGGACCTGGCCGTCCGTCTCCTGCACCGCGGG<br>CTAG

Supplementary Table S2. Oligonucleotide primers used in this study.

Name	Sequence (5′-3′)[a]	PCR-amplified DNA fragments
P01	GGACTCTGGGGTTCGAAGTGGCTCTGCTACGGCCG	*φK38-1 integrase* gene
P02	TGGTCGGTCATTTCGAAAAACGCCCCCAAACGAAAAGC	
P03	GGACTCTGGGGTTCGAATGGCGCCGGACGGGGCTTC	*φBT1 integrase* gene
P04	TGGTCGGTCATTTCGAATAGCCTAACTAACGATTTCAAGGCC	
P05	GACGGCCAGTGAATTGGTACCAGGATCTCGATC	*T7* promoter-MCS-*T7* terminator
P06	TGATTACGCCAAGCTCAGATCTCTCCTTTCAGCA	
P07	GGCCAGTGAATTCGAGCTCGGTACCTCTAGAGTCGACCTGCAGACG	*litR*-*litS*-*crtE*p
P08	TGGTGTTCATATGCCACTGCTTCCGCCG	
P09	GCAGTGGCATATGAACACCATCAACATCGCC	*T7 RNA polymerase* gene (codon usage optimized to *S. coelicolor* A3(2))
P10	GACCATGATTACGCCAAGCTTTCAGGCGAAGGCGAAGTCG	
P11	CTCGAGGGTACCAGGATCTCGATCCCG	*T7* promoter-MCS-*T7* terminator
P12	CTCGAGCAATTGAGATCTCTCCTTTCAGCAAAA	
P13	CTCGAGCATATGAACAAAGGTGTAATGCGAC	*xylE* of *Pseudomonas putida*
P14	CTCGAGAAGCTTTCAGGTCAGCACGGTCATGAATC	
P15	CTCGAGCATATGCTGCGGCCGGTAGAGACC	*gus* (codon usage optimized to *S. coelicolor* A3(2))
P16	CTCGAGAAGCTTTCACTGCTTGCCGCCCTGCTG	
P17	AAGGAGATATACATATGAACAAAGGTGTAATGCGA	*xylE* of *Pseudomonas putida*
P18	TCAGGTCAGCACGGTCATGAATCGGATCCTCACTGCTTGCCGCCCT	
P19	AAGGAGATATACATATGCTGCGGCCGGTAGAGA	*gus* (codon usage optimized to *S. coelicolor* A3(2))
P20	ACCTAAGCTTGGATCCTCACTGCTTGCCGCCCT	
P21	TTAAGAAGGAGATATACATATGGCGACCCTGTGCCGACC	*rppA* of *S. griseus* NBRC 13350
P22	GAGAGACCTAAGCTTGGATCCTCAGCCGGACAGCGCAACGC	
P23	AAGGAGATATACATATGAGACCGAACCGCTTCTC	*LAP* (*SGR_5809*) of *S. griseus* NBRC 13350
P24	CGAGAGACCTAAGCTTTCAGAAGGTGAGCTTCCAGCTG	
P25	AAGGAGATATACATATGAAGCTCGCCCGACTCAGATCC	*LAP* (*Sfla_5137*) of S. pratensis DSM 40990
P26	CGAGAGACCTAAGCTTTCAGAAGGTGAGCTGCCAGCTG	
P27	AAGGAGATATACATATGAACAGTTACCGGCAGCCC	*PAP* (*SGR_0506*) of *S. griseus* NBRC 13350
P28	CGAGAGACCTAAGCTTTCAGATCTCGTCGCGGACCAGC	
P29	AAGGAGATATACATATGAGCGTCAGCTACCGCCAGC	*PAP* (*SLV_33905*) of *S. lividans* 66
P30	CGAGAGACCTAAGCTTTCAGGCCTCGTCGCGGGCGAGC	
P31	AAGGAGATATACATATGCGCATACGCCGGAGAGC	*TGase* of *S. mobaraensis* NBRC 13819

P32	CGAGAGACCTAAGCTTTCACGGCCAGCCCTGCTTTAC	
P33	AAGGAGATATACATATGGACAGGCGAGGCTTTAACCGAC	*SLAC* of *S. coelicolor* A3(2)
P34	CGAGAGACCTAAGCTTTCAGTGCTCGTGTTCGTGTG	
P35	AAGGAGATATACATATGGACAGACGCGGGTTCAACCG	*SLAC* of *Streptomyces* sp. AGRT-94
P36	CGAGAGACCTAAGCTTTCAGTGCTCGTGCGGGTC	
P37	AAGGAGATATACATATGACTCAGGGACCGCTTACTAC	*catalase-peroxidase* of *Streptomyces* sp. No. 565
P38	CGAGAGACCTAAGCTTTCAGCCGCGCAGGGCCTGGAC	
P39	AAGGAGATATACATATGAGATCTCTGAGAACGGGCTCC	*xylanase* of *Streptomyces* sp. AGRT-94
P40	CGAGAGACCTAAGCTTTCATCCCACCGTCACCACG	
P41	CCTCTAGAGTCGACCTGCAGGCATGCCATATGCTGCGG	*gus* (codon usage optimized to *S. coelicolor* A3(2))
P42	TGATTACGCCAAGCTTAGATCCCCGATCCGGTCC	
P43	CCTCTAGAGTCGACCTGCAGACGACAACACCTCACGGG	*litR-litS-crtE*p
P44	ACCGGCCGCAGCATATGCCACTGCTTCCGCCGGGT	
P45	CCTCTAGAGTCGACCTGCAGAGCCCGACCCGAGCACGC	*ermE**p
P46	ACCGGCCGCAGCATATGCGCTGGATCCTACCAACC	
P47	AGTGAATTCGAGCTCGGTACCGCCGGACGACAACACCTCAC	RBS$_{nitA}$/ATG
P48	GGCGATGTTGATGGTGTTCATGTCCGTACCTCCGTTGCTAAGC	
P49	AGTGAATTCGAGCTCGGTACC	RBS$_{nitA}$/GTG
P50	CTTGGCGATGTTGATGGTGTTCACGTCCGTACCTCCGTTGCTAAGC	
P51	AGTGAATTCGAGCTCGGTACC	RBS$_{nitA}$/TTG
P52	CTTGGCGATGTTGATGGTGTTCAAGTCCGTACCTCCGTTGCTAAGC	
P53	AGTGAATTCGAGCTCGGTACC	RBS$_{w52}$/ATG
P54	GGCGATGTTGATGGTGTTCATATGTATTATCCTCCAAAAAAAATAAATAAG	
P55	AGTGAATTCGAGCTCGGTACC	RBS$_{sav2794}$/ATG
P56	GGCGATGTTGATGGTGTTCATATGTGACTCCTTCTGCGTAAGCTTG	
P57	GACGGCCAGTGAATTCTAATACGACTCACTATAGGGAGACCACAACGG TAAGAAGGAGATATACATATG	Trimming of *T7* promoter
P58	GACGGCCAGTGAATTCTAATACGACTCACTATAGGGAGACCACAACGA AGAAGGAGATATACATATG	
P59	GACGGCCAGTGAATTCTAATACGACTCACTATAGGGAGACCACAAAAG AAGGAGATATACATATG	
P60	GACGGCCAGTGAATTCTAATACGACTCACTATAGGGAGACCACAAGAA GGAGATATACATATG	
P61	GACGGCCAGTGAATTCTAATACGACTCACTATAGGGAGACCAAGAAGG AGATATACATATG	

P62	TGATTACGCCAAGCTTTCACTGCTTGCCGCCCTG

[a] Restriction enzyme sites were underlined.

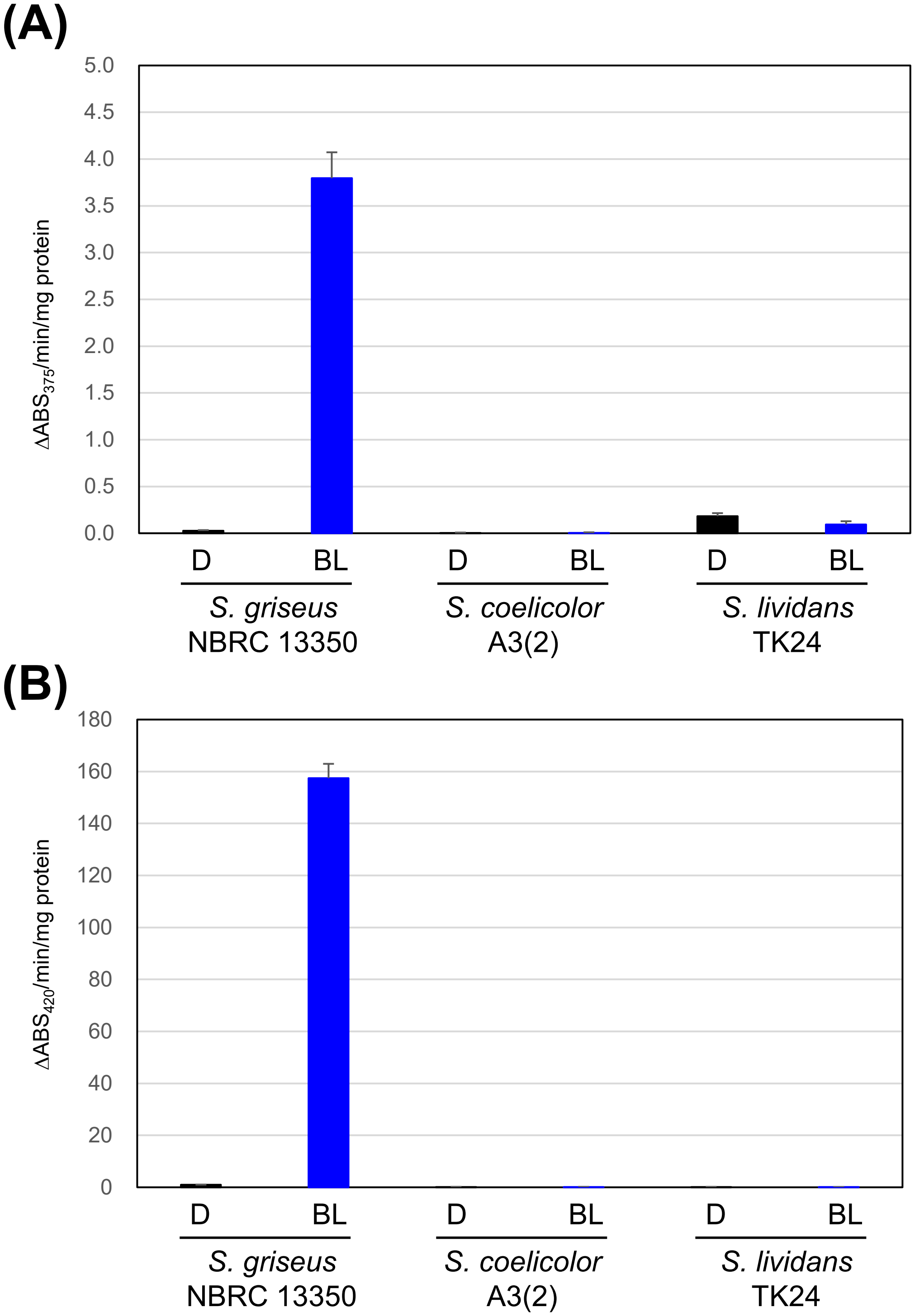

Supplementary Figure S1. Functionality of the multicopy plasmid type pLit19 in *S. griseus*, *S. coelicolor*, and *S. lividans*

S. griseus, S. coelicolor A3(2)*,* and *S. lividans* TK24 transformants harboring pLit19-XylE (A) or pLit19-GUS (B) were cultured with shaking for 48 h under dark (D) or blue light (BL; 3 $\mu mol \cdot s^{-1} \cdot m^{-2}$). Enzymatic activities of XylE and GUS in the cell-free extracts were calculated as the change in absorbance (ABS) at 375 and 420 nm per minute per milligram of total protein (ΔABS375 nm/min/mg or ΔABS420 nm/min/mg), respectively. Data are represented as mean ± standard deviation (SD) from three independent experiments (*n* = 3).

Supplementary Figure S1

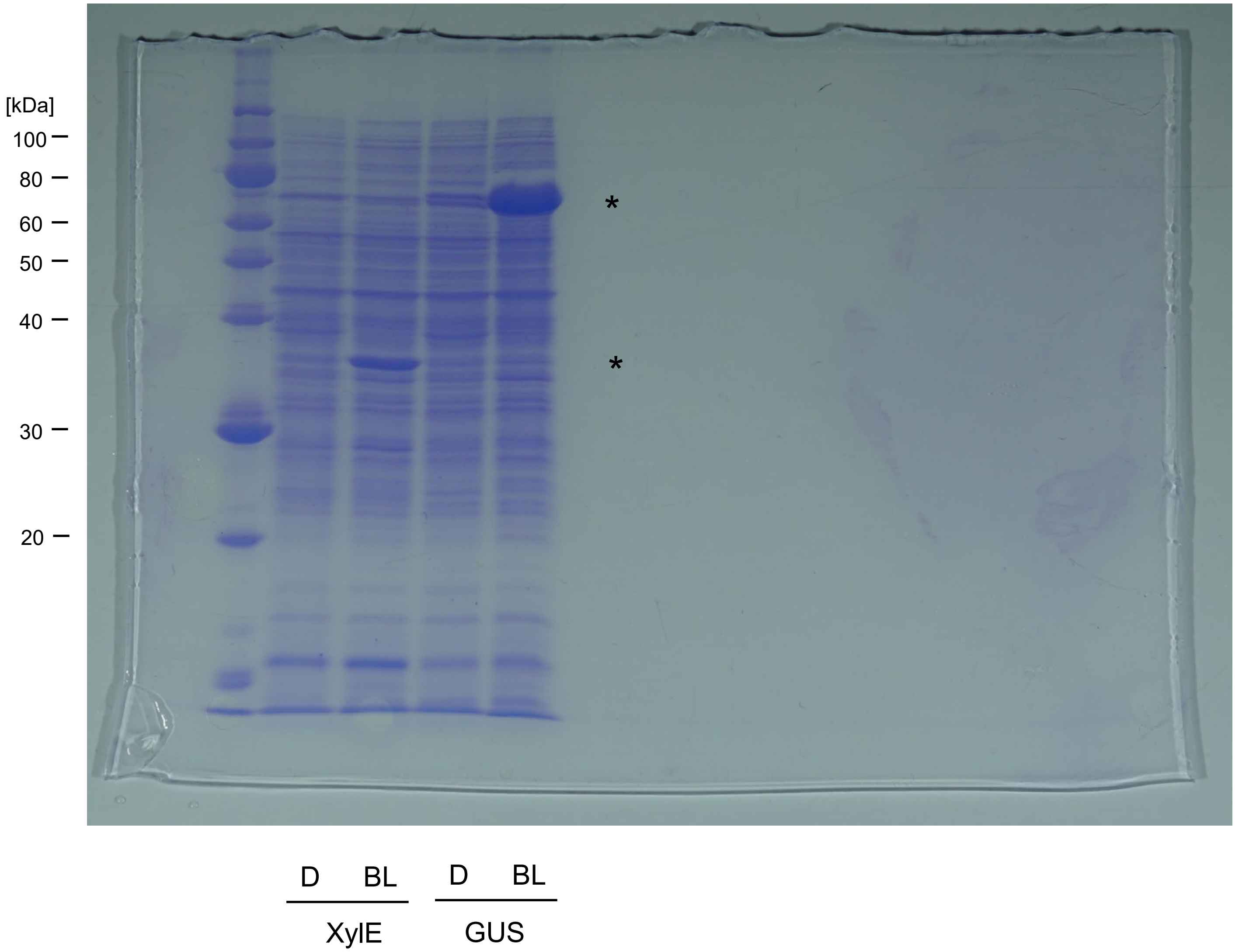

Supplementary Figure S2
The full-length, unprocessed gel image of Figure 2.

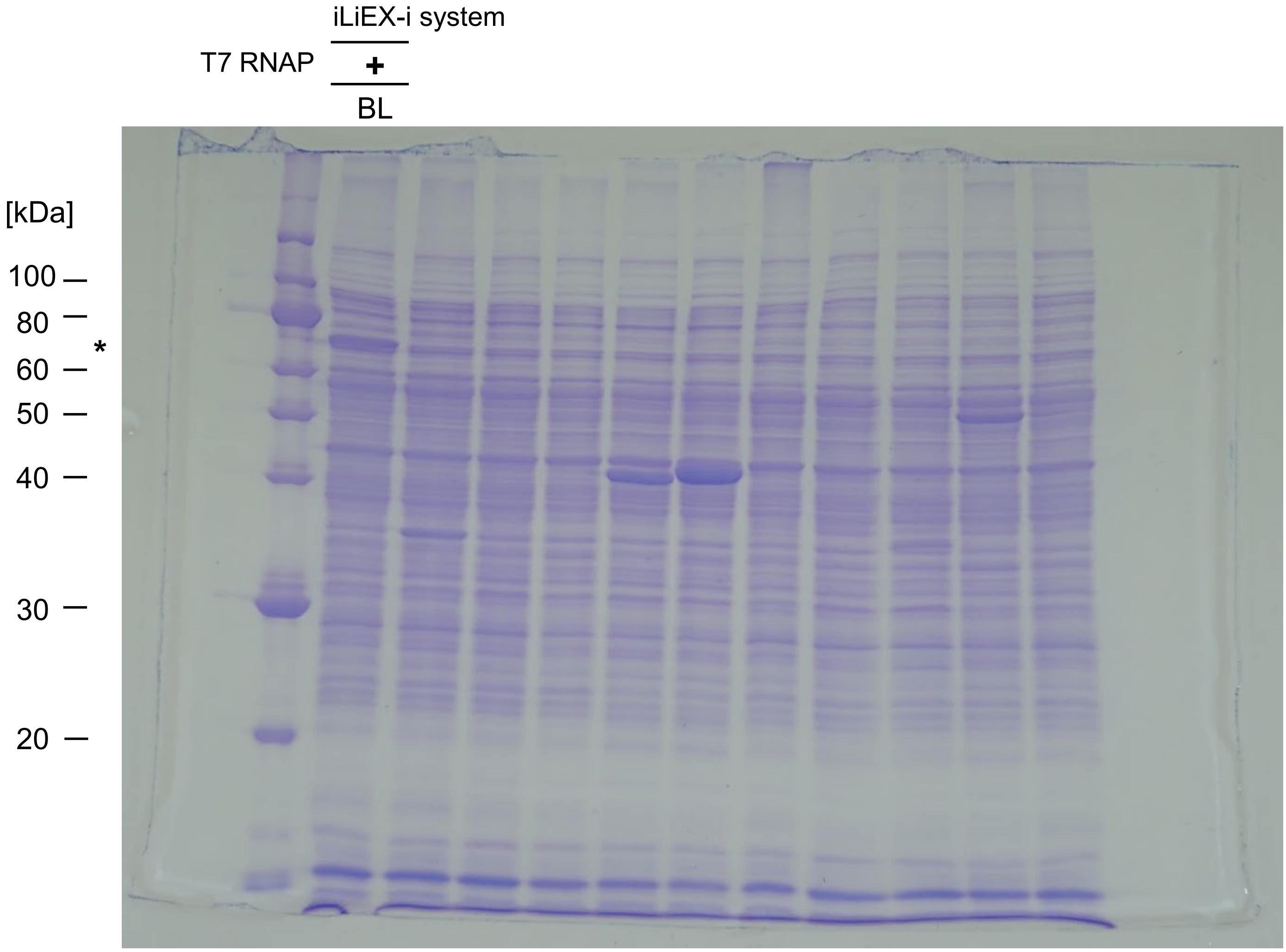

Supplementary Figure S3
The full-length, unprocessed gel image of Figure 3.

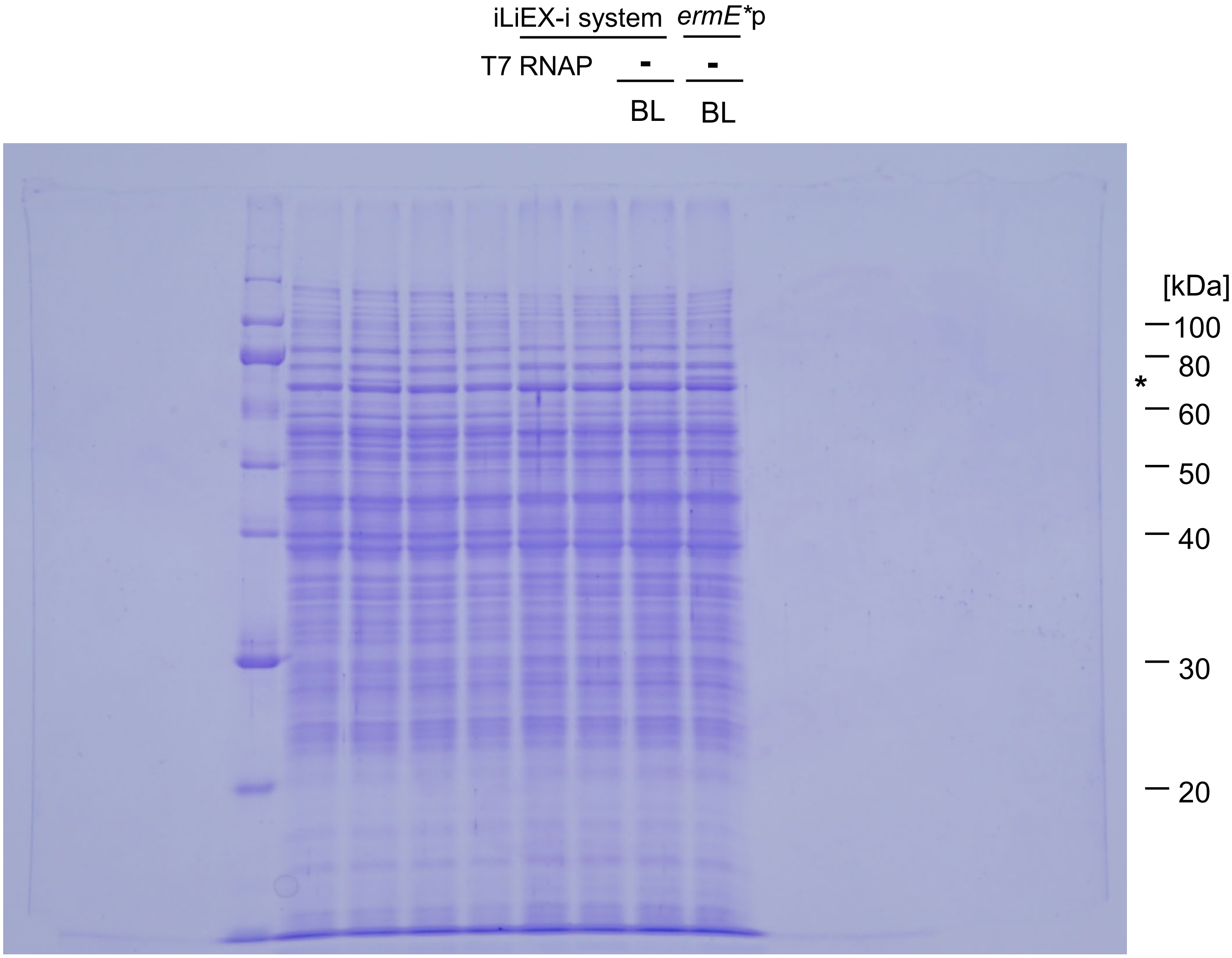

Supplementary Figure S4
The full-length, unprocessed gel image of Figure 3.

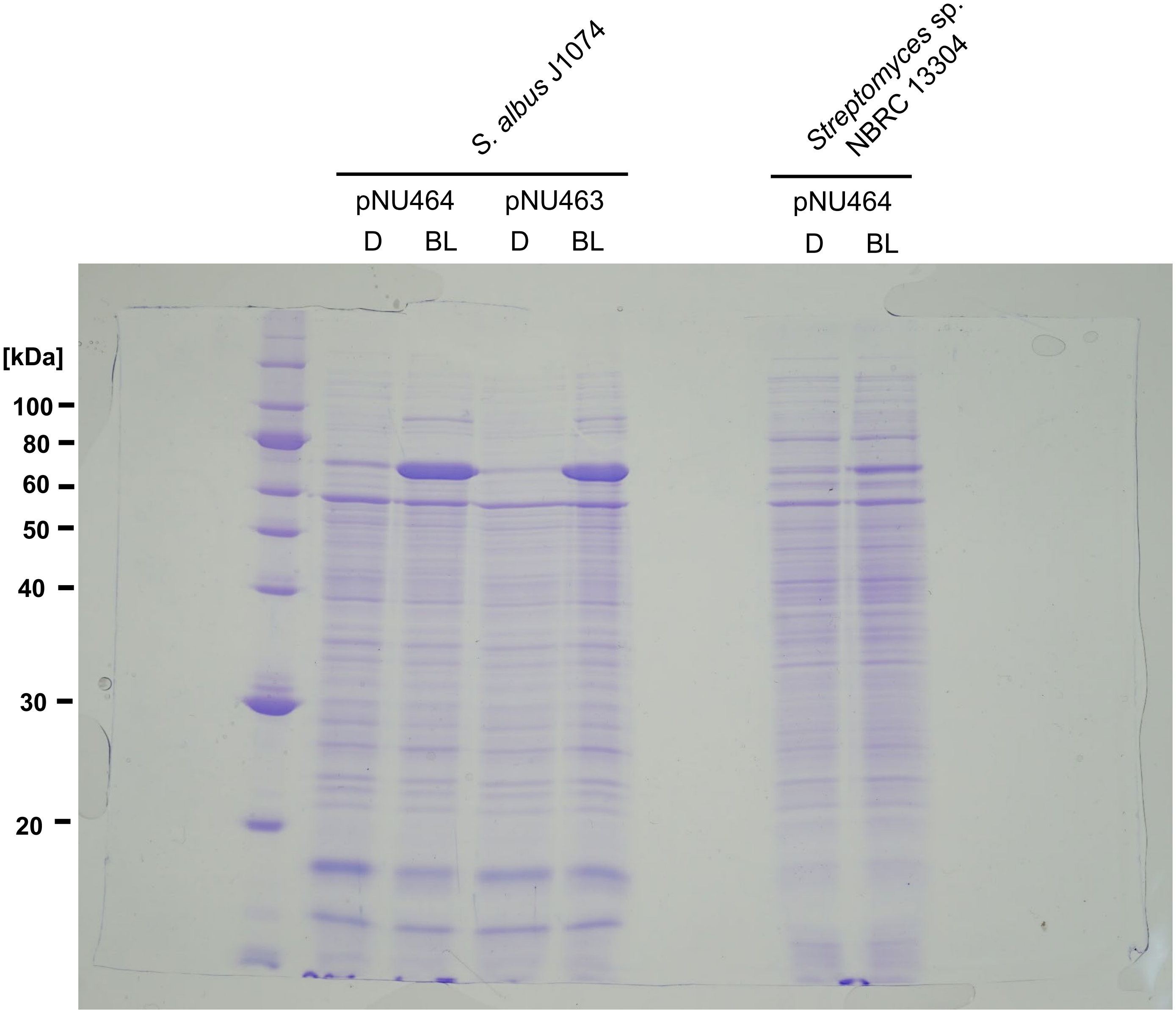

Supplementary Figure S5
The full-length, unprocessed gel image of Figure 4.

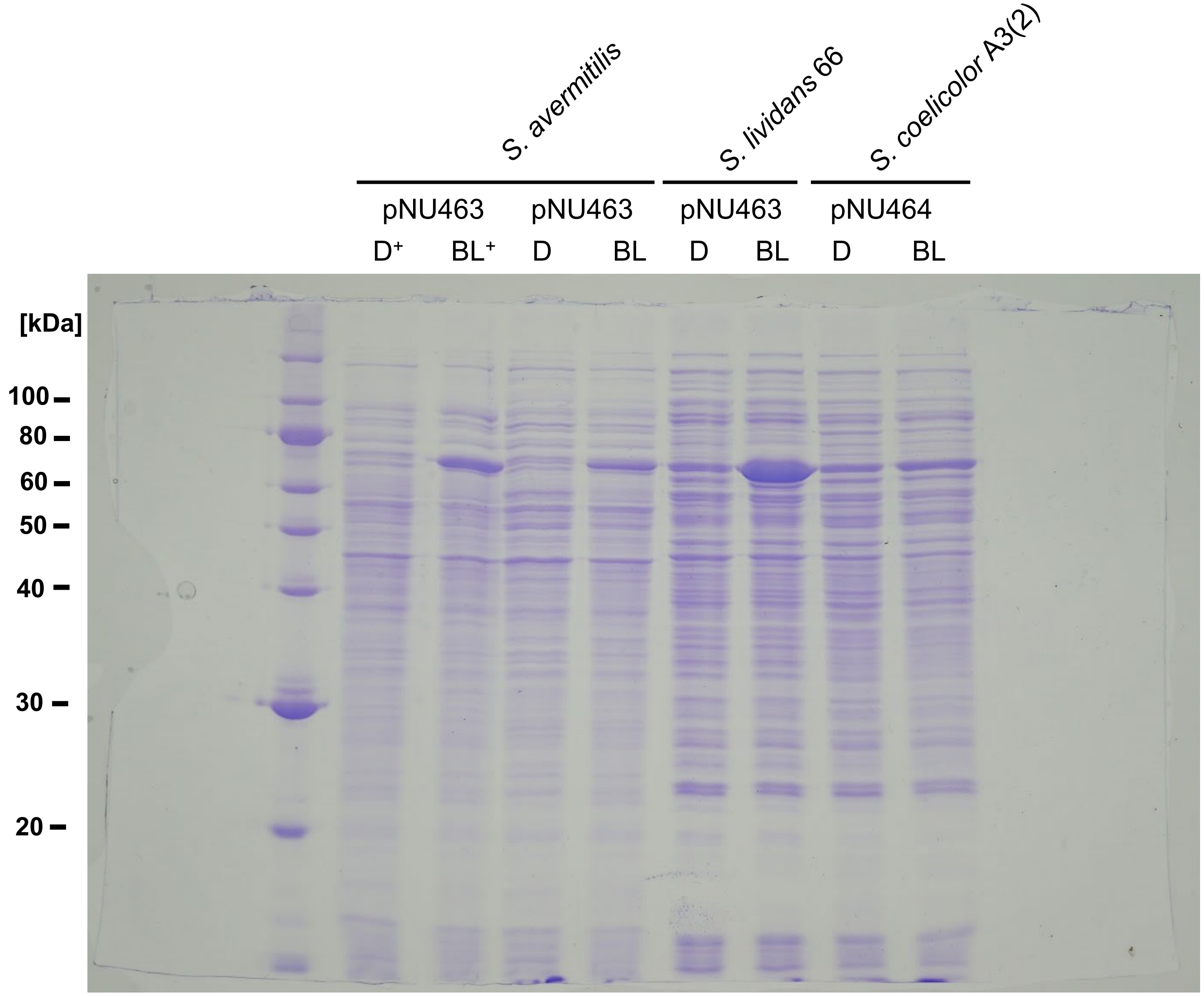

Supplementary Figure S6
The full-length, unprocessed gel image of Figure 4.

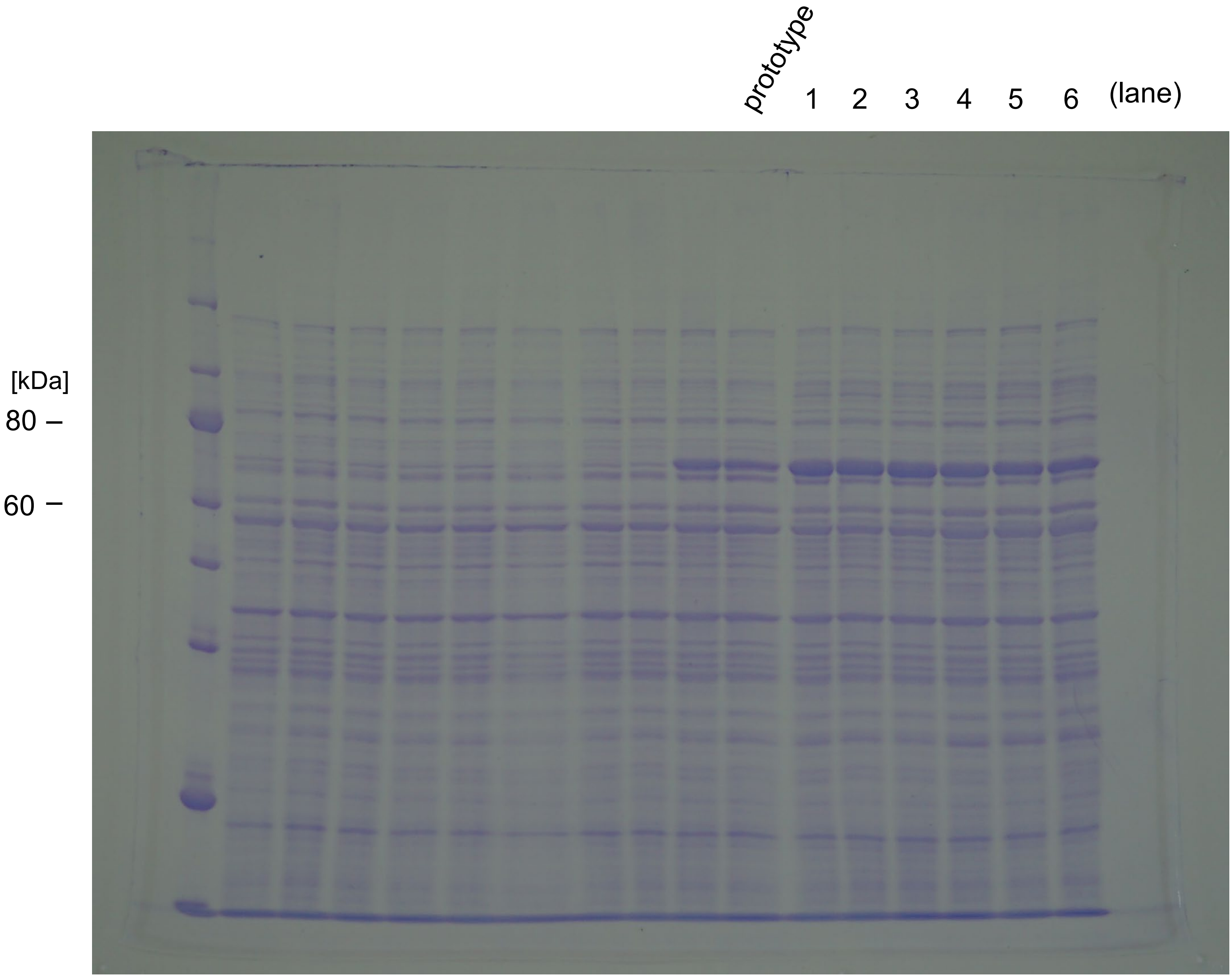

Supplementary Figure S7
The full-length, unprocessed gel image of Figure 5.

(A)

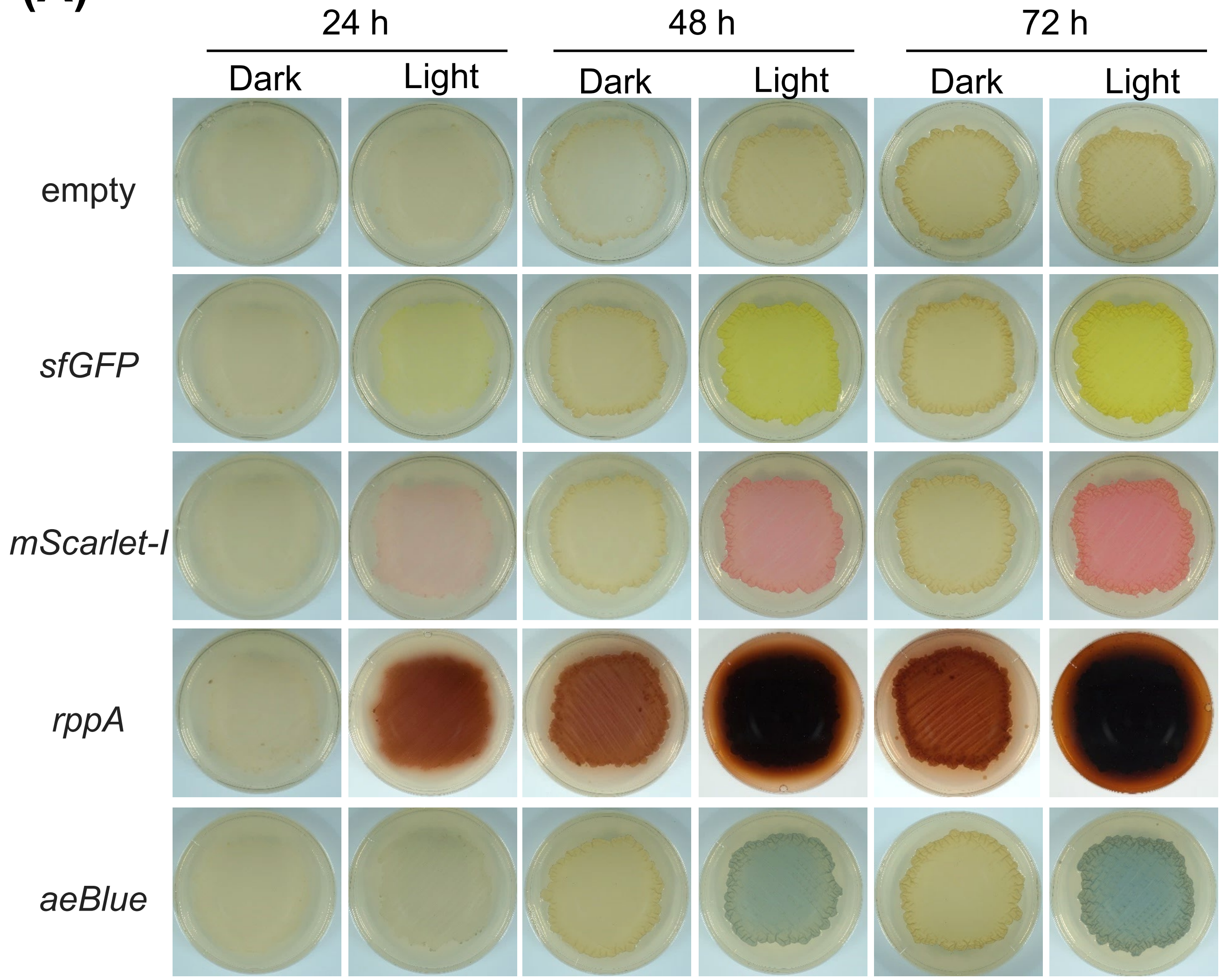

(B)

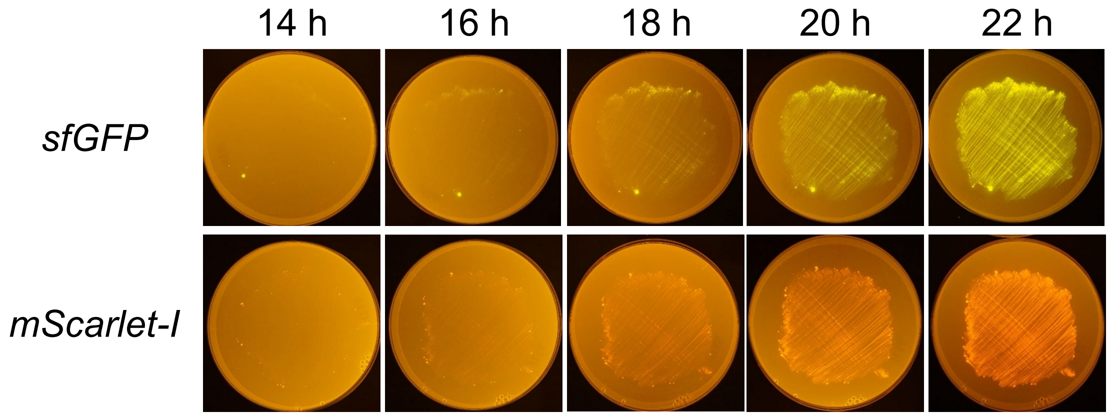

Supplementary Figure S8
The full-length, unprocessed gel images of Figure 8.

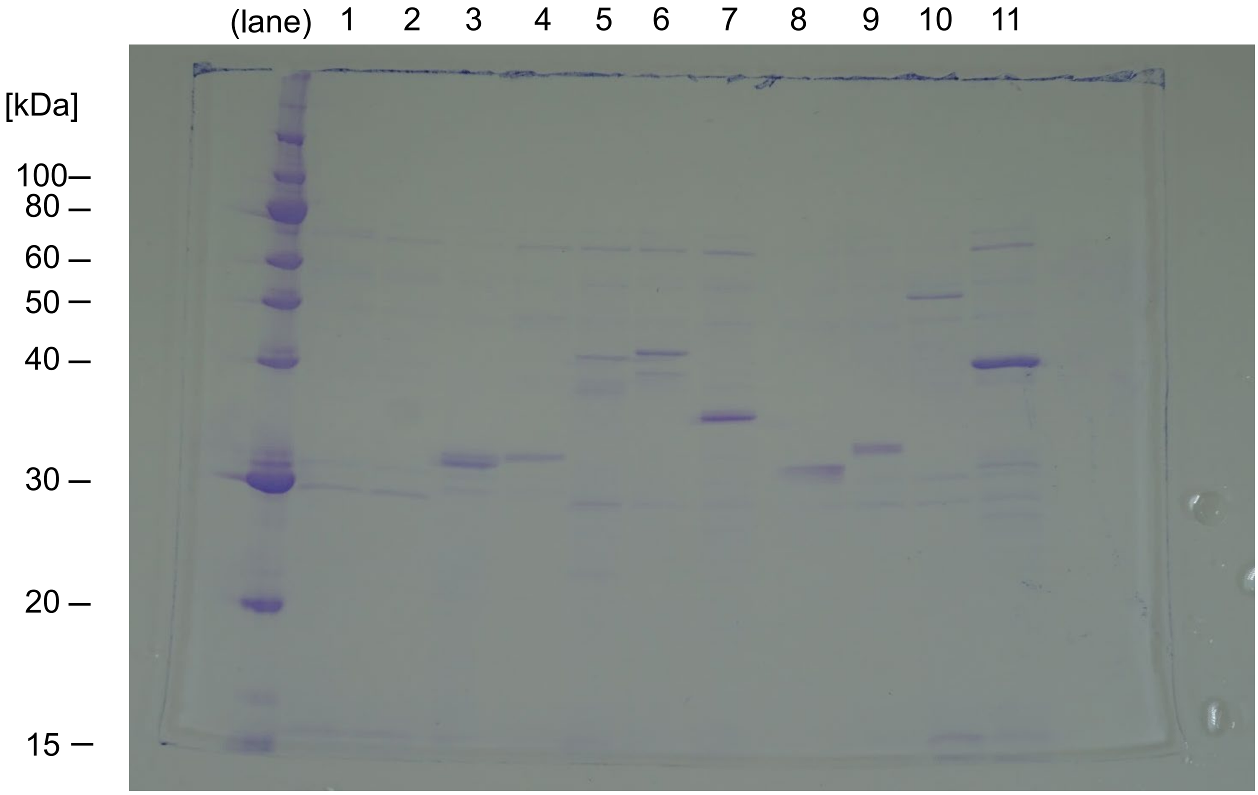

Supplementary Figure S9
The full-length, unprocessed gel image of Figure 9.

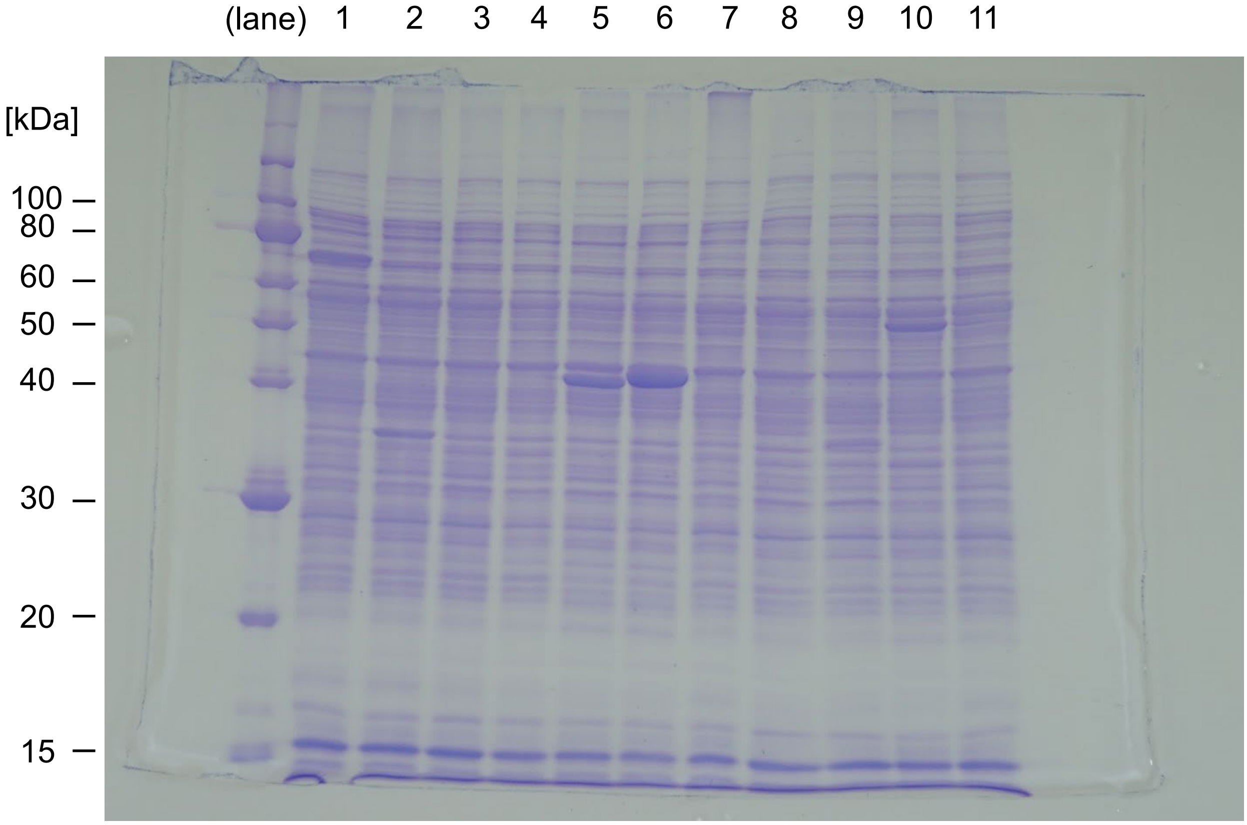

Supplementary Figure S10
The full-length, unprocessed gel image of Figure 9.

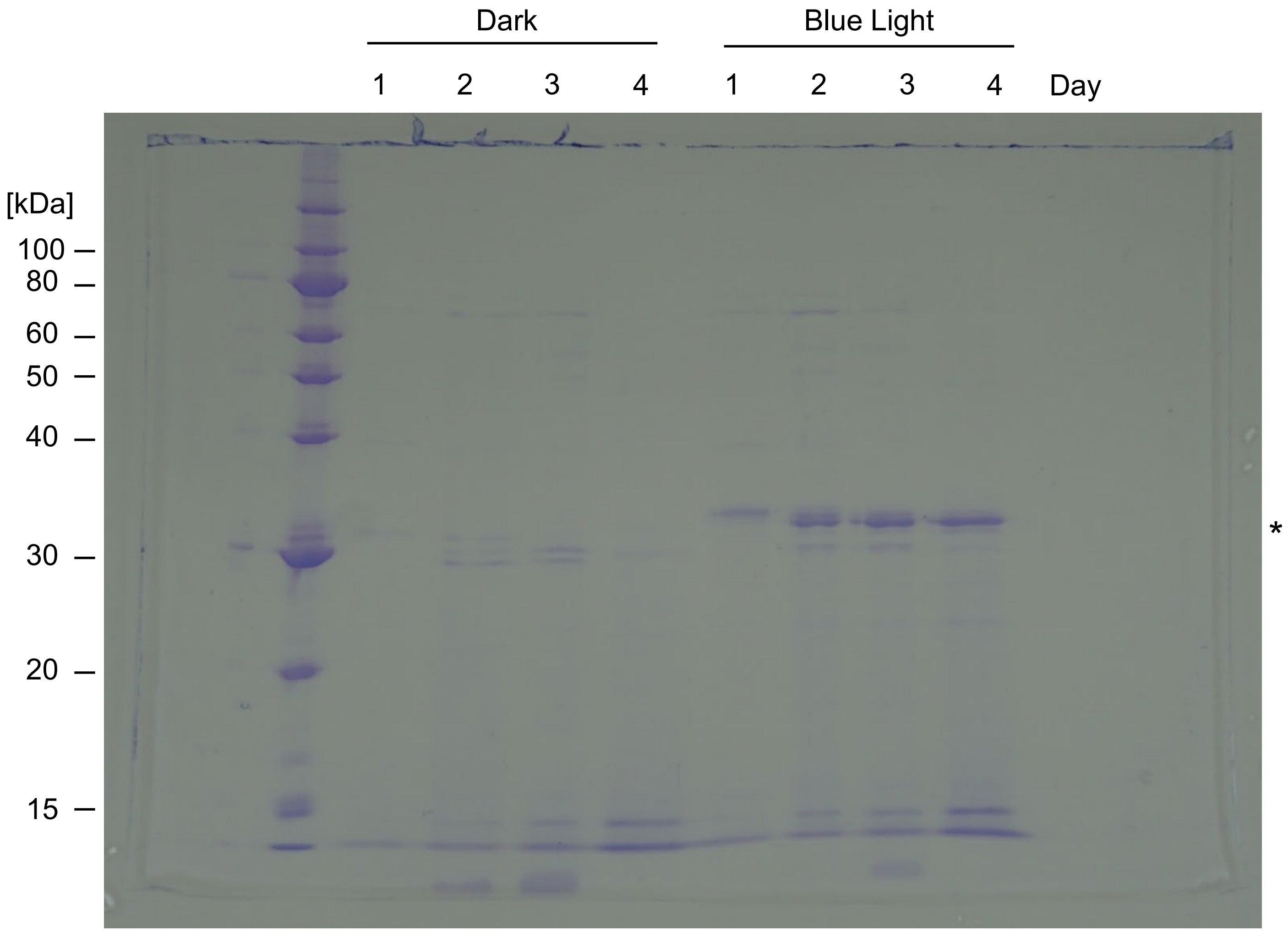

Supplementary Figure S11
The full-length, unprocessed gel image of Figure 10.
